# Differential Metabolic Reprogramming in *Paenibacillus* *alvei*-Primed *Sorghum* *bicolor* Seedlings in Response to *Fusarium pseudograminearum* Infection

**DOI:** 10.3390/metabo9070150

**Published:** 2019-07-23

**Authors:** René Carlson, Fidele Tugizimana, Paul A. Steenkamp, Ian A. Dubery, Nico Labuschagne

**Affiliations:** 1 Department of Plant and Soil Sciences, Faculty of Plant Pathology, University of Pretoria, Private Bag X20, Hatfield, Pretoria 0028, South Africa; 2 Centre for Plant Metabolomics Research, Department of Biochemistry, Faculty of Science, University of Johannesburg, P.O. Box 524, Auckland Park, Johannesburg 2006, South Africa

**Keywords:** crown rot, *Fusarium pseudograminearum*, induced systemic resistance, LC-MS, *Paenibacillus alvei*, PGPR, phytoalexin, priming, *Sorghum bicolor*

## Abstract

Metabolic changes in sorghum seedlings in response to *Paenibacillus alvei* (NAS-6G6)-induced systemic resistance against *Fusarium pseudograminearum* crown rot were investigated by means of untargeted ultra-high performance liquid chromatography-high definition mass spectrometry (UHPLC-HDMS). Treatment of seedlings with the plant growth-promoting rhizobacterium *P. alvei* at a concentration of 1 × 10^8^ colony forming units mL^−1^ prior to inoculation with *F. pseudograminearum* lowered crown rot disease severity significantly at the highest inoculum dose of 1 × 10^6^ spores mL^−1^. Intracellular metabolites were subsequently methanol-extracted from treated and untreated sorghum roots, stems and leaves at 1, 4 and 7 days post inoculation (d.p.i.) with *F. pseudograminearum*. The extracts were analysed on an UHPLC-HDMS platform, and the data chemometrically processed to determine metabolic profiles and signatures related to priming and induced resistance. Significant treatment-related differences in primary and secondary metabolism post inoculation with *F. pseudograminearum* were observed between *P. alvei*-primed versus naïve *S. bicolor* seedlings. The differential metabolic reprogramming in primed plants comprised of a quicker and/or enhanced upregulation of amino acid-, phytohormone-, phenylpropanoid-, flavonoid- and lipid metabolites in response to inoculation with *F. pseudograminearum*.

## 1. Introduction

As an evolutionary adaptation to survive a sessile existence, in addition to pre-existing physical and chemical barriers, plants have developed the ability to actively protect themselves against environmental stress through the synthesis of complex and ever-changing mixtures of defence-related metabolites [1]. These metabolites can either be synthesised locally or systemically as part of systemic acquired resistance (SAR) [2,3]. SAR has an associated fitness cost that has a negative impact on the plant’s growth and development, but also indirectly impacts its microbiome [4]. It is hypothesised that in response to the negative ecological impact of SAR, evolutionary forces were directed towards the development of induced systemic resistance (ISR) [5]. During ISR, the plant is primed by beneficial microbes for an enhanced defensive response that only activates upon challenge with a stress, thus wasting no resources.

During the early stages of priming, an increase in the sensitivity to the signalling hormones jasmonic acid or ethylene [6] facilitates the reprogramming of the plant’s metabolome for enhanced defence [7,8,9]. Upon exposure to a stress, the synthesis of defence metabolites usually occur earlier and in higher quantities in primed compared with naïve plants [10], giving rise to the synthesis of secondary defence metabolites which include phenylpropanoids, terpenoids, polyketides and alkaloids [11].

Plant growth-promoting rhizobacteria (PGPR) are beneficial bacteria occurring in the root zone of plants, promoting plant growth directly through increased uptake of nutrients (biofertilisers), stimulation of plant growth through the production of phytohormones (biostimulants) or through the degradation of organic pollutants (rhizoremediators). PGPR also offer indirect plant growth promotion by protection against biotic- and abiotic stress (bioprotectants) [12]. PGPR can employ either one of these mechanisms or more than one, simultaneously [13,14,15,16]. In addition to this, PGPR are known to induce resistance systemically, giving rise to physical and/or chemical defence responses upon challenge with an external stress [17,18].

Sorghum [*Sorghum bicolor* (L.) Moench] is an agricultural grain crop of significant importance for food security and sustainable livelihoods in developing countries [19]. In the current study, we investigated the protection offered by a PGPR, *P. alvei*, in mitigating the disease susceptibility of sorghum seedlings towards *F. pseudograminearum,* the causative agent of crown rot disease. During a time course study, we employed an untargeted ultra-high performance liquid chromatography-high definition mass spectrometry (UHPLC-HDMS)-based metabolomics approach to compare the adaptive metabolic changes that result in the altered metabolomes upon challenge with a biotic stress in primed versus naïve sorghum seedlings.

## 2. Results

### 2.1. Plant Growth Parameters and Crown Rot Disease Severity

**Figure 1:**
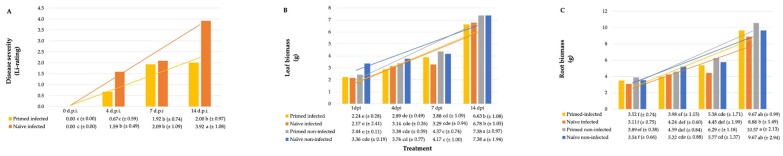
Effect of *P. alvei* alone and in combination with *F. pseudograminearum* on mean plant biomass and crown rot disease severity. (**A**) Crown rot disease severity and (**B**,**C**) leaf- and root biomass at 1, 4, 7 and 14 d.p.i. with *F. pseudograminearum* in *P. alvei*-primed versus naïve *S. bicolor* seedlings. Means followed by the same letter does not differ significantly according to Tukey’s LSD test at a significance level of *p* < 0.05. Numbers in parenthesis are the standard deviation from the mean. (**A**) Disease severity calculated according to a ‘0–5 scale’ based on lesion severity [21]. Legend: Primed: Primed with *P. alvei*; Naïve (no priming); Infected: infected with *F. pseudograminearum*; Non-infected: not infected with *F. pseudograminearum.*

The initial inoculum-dose study was aimed at optimising conditions for *P. alvei*-induced systemic resistance against crown rot. Crown rot disease severity increased significantly with increment in the inoculum dose (Table 1). Treatment with *P. alvei* caused a significant reduction in disease severity and an increase in root and shoot weights at the higher inoculum dose of 1 × 10^6^ spores mL^−1^ (Table 1). Therefore, this inoculum dose was used in the time course study and it was found that disease severity increased as time progressed post inoculation (Figure 1A). However, the rate of disease progression was significantly lower in *P. alvei*-primed *S. bicolor* seedlings. This trend was correspondingly reflected in the fresh leaf- and root biomass of the seedlings (Figure 1B,C respectively).

To confirm that *F. pseudograminearum* was in fact the causal organism in both the inoculum dose- and time course studies, isolations were made from crown rot lesions on the sorghum seedlings. Excised, surface-sterilised stem segments were plated onto rose bengal-glycerol-urea (RbGU) medium [20] and incubated under near-UV light to induce spore formation. *F. pseudograminearum* growth was identified morphologically by means of microscopy (Figure S1).

### 2.2. Metabolomic Profiles of *Paenibacillus alvei*-Primed and Naïve *Fusarium pseudograminearum* Infected Sorghum Plants

**Figure 2:**
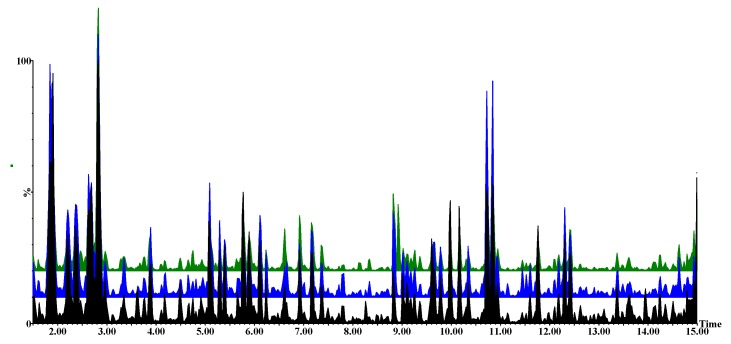
Untargeted ultra-high performance liquid chromatography-high definition mass spectrometry (UHPLC-HDMS) base peak intensity (BPI) chromatograms of electrospray ionisation (ESI)-positive data indicating the metabolomic profiles of untreated (black), naïve infected (blue) and primed infected (green) roots obtained at 1 d.p.i. with *F. pseudograminearum*.

Visual inspection of the UHPLC-HDMS base peak intensity (BPI) chromatograms showed evidently differential peak population (presence, intensities) of *P. alvei*-primed- and naïve *F. pseudograminearum* infected *S. bicolor* seedlings versus the untreated controls. The chromatographically distinct BPI chromatograms of these three treatments for the electrospray ionisation (ESI)-positive data at 1 day post inoculation (d.p.i.) with *F. pseudograminearum* taken from root samples are shown in Figure 2 and those for stem- and leaf samples are provided in the supplementary material as Figures S2 and S3. These chromatographic differences reflect differential metabolite profiles (and composition) in samples derived from *P. alvei*-primed- and naïve *F. pseudograminearum* infected *S. bicolor* seedlings versus the untreated controls.

**Table 1:** Effect of *P. alvei* alone and in combination with three dose levels of *F. pseudograminearum* on mean ^1^ mass and disease severity of *S. bicolor* seedlings at 14 days post inoculation (d.p.i.).

Treatment		Crown Rot				Fresh Mass				Dry Mass			
*P. alvei*	*F. pseudograminearum*	Li-Rating ^3^		Isolations ^4^		Leaves		Roots		Leaves		Roots	
(cfu mL^−1^) ^2^	(spores mL^−1^)			(%)		(g)		(g)		(g)		(g)	
0	0	0.00 (±0.00)	e	0.00 (±0.00)	c	0.68 (±0.16)	c	0.73 (±0.30)	a	0.14 (±0.07)	a	0.04 (±0.01)	ab
1 × 10^8^	0	0.00 (±0.00)	e	0.00 (±0.00)	c	0.75 (±0.21)	ab	0.80 (±0.35)	a	0.14 (±0.06)	a	0.05 (±0.02)	a
0	1 × 10^2^	1.14 (±1.03)	cd	22.67 (±8.84)	ab	0.65 (±0.21)	c	0.74 (±0.36)	a	0.11 (±0.07)	b	0.05 (±0.02)	a
0	1 × 10^4^	1.90 (±0.96)	b	24.00 (±9.66)	ab	0.67 (±0.18)	c	0.68 (±0.28)	a	0.10 (±0.05)	b	0.05 (±0.02)	a
0	1 × 10^6^	3.06 (±1.80)	a	26.00 (±6.99)	a	0.55 (±0.11)	d	0.55 (±0.19)	a	0.06 (±0.02)	c	0.04 (±0.02)	ab
1 × 10^8^	1 × 10^2^	0.57 (±0.86)	de	21.33 (±10.60)	ab	0.58 (±0.19)	d	0.65 (±0.32)	a	0.10 (±0.07)	b	0.03 (±0.00)	b
1 × 10^8^	1 × 10^4^	1.32 (±1.05)	bc	21.00 (±11.01)	ab	0.70 (±0.24)	bc	0.78 (±0.32)	a	0.11 (±0.06)	b	0.05 (±0.03)	a
1 × 10^8^	1 × 10^6^	1.67 (±1.35)	bc	18.00 (±13.17)	b	0.76 (±0.18)	a	0.81 (±0.38)	a	0.11 (±0.07)	b	0.05 (±0.03)	a

^1^ Means within columns followed by the same letter does not differ significantly according to Tukey’s least significance determination (LSD) test at a significance level of *p* < 0.05. Numbers in parenthesis are the standard deviation from the mean. ^2^ Colony forming units per millilitre. ^3^ Disease severity rating calculated according to a ‘0-5 scale’ based on lesion severity [21]. ^4^ Percentage of the isolations made from the stem area that yielded growth on *Fusarium*-selective medium.

In order to elucidate informative description of specific metabolic features related to these observed differential chromatographic profiles, data mining and comparative chemometric analyses were performed as described in the experimental section. Chemometric analyses employed included unsupervised methods, such as principal component analysis (PCA), hierarchical clustering analysis (HCA) and a supervised approach, namely orthogonal partial least square-discriminant analysis (OPLS-DA).

**Figure 3:**
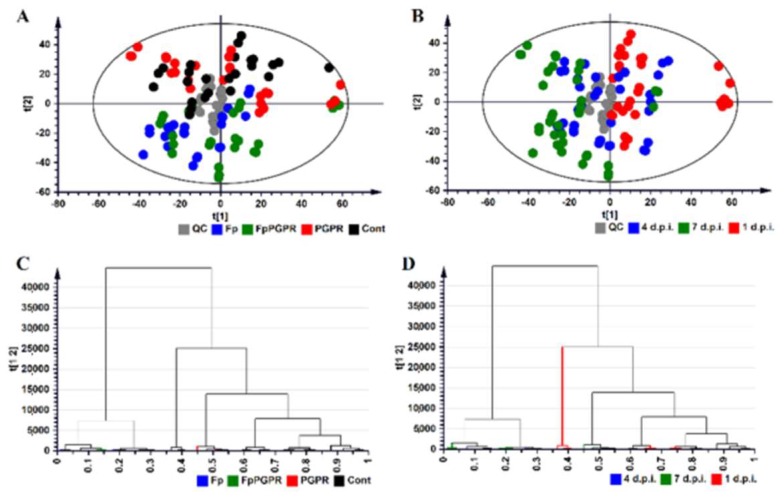
Principal component analysis (PCA) score/scatter plot of root samples computed from ESI-positive data representing the first two PCs of a 17-component PCA model. The model explains 74.3% variation in the Pareto-scaled data (the total amount of explained variation in X; R^2^X = 0.743) and 56.6% predicted variation according to cross-validation (the total amount of predicted variation; Q^2^ = 0.566). (**A**,**B**) represents the same PCA scores plot with (**A**) showing the treatment-related clustering and (**B**) showing the time-related clustering. (**C**,**D**) Hierarchical clustering analysis (HCA) dendrograms corresponding to (**A**,**B**). Legend: QC: Quality control (grey); Fp: Naïve plants inoculated with *F. pseudograminearum* (blue); FpPGPR: *P. alvei*-primed plants inoculated with *F. pseudograminearum* (green); PGPR: *P. alvei*-primed plants (red); Cont: Untreated plants (black); 1 d.p.i.: 1 d.p.i. with *F. pseudograminearum* (red); 4 d.p.i.: 4 d.p.i. with *F. pseudograminearum* (blue); 7 d.p.i.: 7 d.p.i. with *F. pseudograminearum* (green).

The PCA and HCA computed for the root samples of the ESI-positive data are shown in Figure 3. The remaining models computed for the ESI-positive data (stem- and leaf samples) and ESI-negative data (root-, stem- and leaf-samples) are provided in the supplementary material as Figures S4–S8. The close clustering of the quality control (QC) samples in the PCA, indicate that the UHPLC-HDMS system was stable and that results were reproducible. The PCA models provide evidence for both treatment-related clustering (Figure 3A) and time-related clustering (Figure 3B).

**Figure 4:**
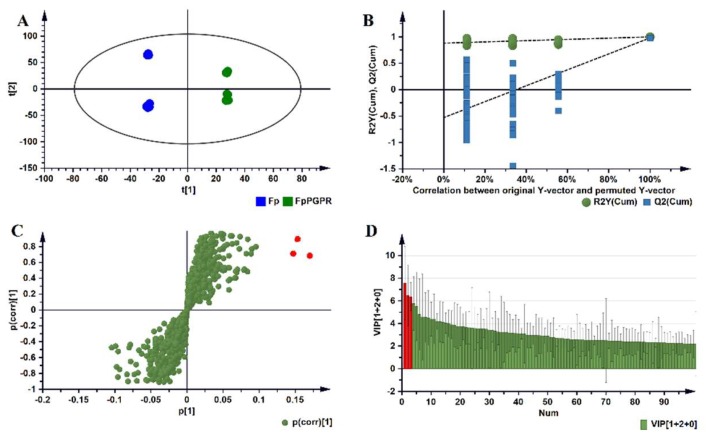
Orthogonal partial least square-discriminant analysis (OPLS-DA) modelling and variable/feature selection ESI-positive data (roots samples). (**A**) A typical PCA scores/scatter plot for the OPLS-DA model separating naïve (Fp) versus primed plants (FpPGPR) at 1 d.p.i. (1 + 2 + 0 components, R^2^X = 0.630, Q^2^ = 0.980, CV-ANOVA, cross-validated analysis of variance, *p*-value < 0.05). In the scores plot, it is evident that the two groups are clearly separated: naïve versus primed. (**B**) A typical response permutation test plot (n = 100) for the OPLS-DA model in (**A**); the R^2^ and Q^2^ values of the permutated models correspond to y-axis intercepts: R^2^ = (0.0, 8.90) and Q^2^ = (0.0, 0.542). (**C**) An OPLS-DA loadings S-plot for the same model in (**A**); variables situated in the extreme end of the S-plot are statistically relevant and represent leading candidates as discriminating variables/features. (**D**) Variable importance for the projection (VIP) plot for the same model; pointing mathematically to the importance of each variable (feature) in contributing to group separation in the OPLS-DA model. (**C**,**D**) Examples of the variables that were significantly upregulated in primed plants are highlighted in red.

**Figure 5:**
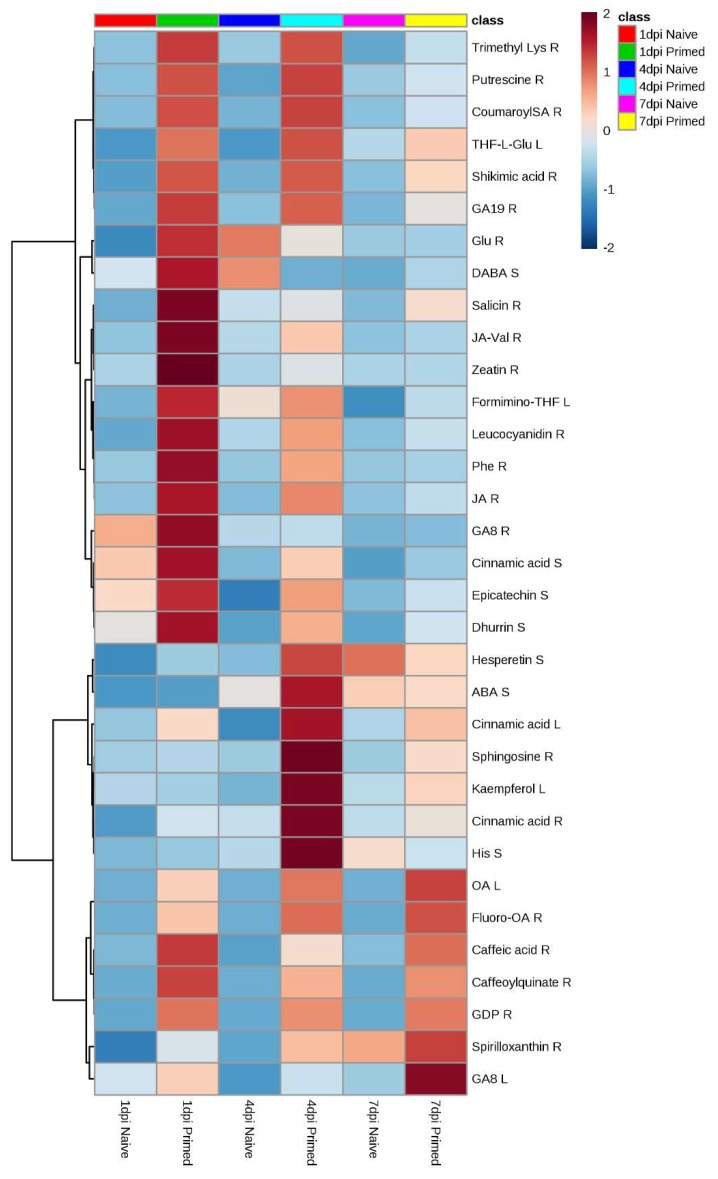
Heatmap showing the top 33 metabolites (abbreviations listed in Table 2 under the heading “Heatmap key”) upregulated in roots (R), stems (S) and leaves (L) of *P. alvei*-primed and naïve *S. bicolor* seedlings at 1, 4 and 7 d.p.i. with *F. pseudograminearum*

The OPLS-DA computed for the root samples of the ESI-positive data is shown in Figure 4. Those for stem- and leaf samples are provided in the supplementary material as Figures S9 and S10 and a summary of the description and validation of all the computed OPLS-DA models is given in Table S1. All the OPLS-DA models used in this study were perfect binary classifiers and had no signs of possible overfitting, as indicated by cross-validation, and none of the permutated models (n = 100) performed better than the original models in separating classes, as typically shown by Figure 4B. For selection of ‘variables’, i.e., discriminating metabolite features with unique Rt-*m/z* values, OPLS-DA loadings S-plots (Figure 4C) were evaluated: these loading plots aid in identifying variables which differ between groups, i.e., the discriminating features. Variables that combine high model influence (covariation) with high reliability (correlation), i.e., variables at the far ends of the S-plot, are statistically relevant as potential discriminant variables to be selected [22]. To avoid bias in the selection of variables, the variable importance in projection (VIP)-plots were generated (Figure 4D) and only the variables (from S-plots) with VIP scores >1.0 were retained. Examples of variables that were significantly upregulated and identified as biomarkers in primed plants are highlighted in red. As mentioned in the experimental section, 41 statistically selected variables (from S-plots) were then annotated to MSI level-2 and are reported in Table 2, consisting of a total of 37 metabolites from the amino acid-, phytohormone-, flavonoid-, phenylpropanoid- and lipid metabolite class. To aid in the visualisation of the differential metabolic programming of primed plants compared with those left naïve, heatmap analysis was performed (Figure 5). In addition to this metabolic pathway- and Venn diagram- analyses are provided in the supplementary material as Figures S11 and S12.

## 3. Discussion

During the ISR response, specific consecutive steps are necessary for a successful tripartite interaction, namely (1) an initial plant-microbe recognition, (2) the activation of downstream signal transduction pathways during priming and, finally, (3) the defence response upon challenge with a pathogen [23]. In the current study a similar interaction between *P. alvei*, *S. bicolor* and *F. pseudograminearum* gave rise to ISR. Priming *S. bicolor* seedlings with *P. alvei* resulted in an early, enhanced upregulation of both primary and secondary metabolism upon inoculation with *F. pseudograminearum* as shown in the pathway analysis (provided in the supplementary material as Figure S11). Pathways involved in phytohormone-, amino acid- and flavonoid- metabolism were affected. 

In the current study, the early upregulation of jasmonic acid (JA) (28 × change, Table 2) in *P. alvei*-primed roots at 1 d.p.i. with *F. pseudograminearum* supports previous findings of its requirement in the initial signal transduction during ISR, giving rise to a reprogramming of the plant’s metabolome for enhanced defence. Although JA or ethylene is required in ISR, other phytohormones also play a role in signal transduction for enhanced defence [23,24]. In the current study zeatin (96 × change), salicin (15 × change) and gibberellin (2 × change) were upregulated in the roots of primed *S. bicolor* seedlings (Table 2). These phytohormones play an important role in plant growth, but also in the protection against stress. When secreted by rhizobacteria for instance, these phytohormones also act as signalling factors that elicit plant defences [25,26].

In addition to the upregulation of phytohormones in primed plants, the changes in primary metabolism also included the group of amino acids, which serve as building blocks for secondary metabolism. Priming thus preconditions the plant for enhanced defence by elevating the amino acid levels that are at the disposal of the plant. In the current study, amino acids and derivatives were upregulated as early as 1 d.p.i. with *F. pseudograminearum*, including phenylalanine (33 × change), glutathione (6 × change), lysine (6 × change), putrescine (4 × change) and histidine (3 × change) (Table 2). Aromatic amino acids such as phenylalanine, that derive from the shikimate pathway, enable the early upregulation of the phenylpropanoid pathway and resultant phenylpropanoid defence metabolites, including phytoalexins, cell wall reinforcements and wound response [27,28]. Glutathione is a tripeptide thiol (consisting of glycine, glutamate and cysteine) that has powerful antioxidant capacity thus offering protection against oxidative stress [29]. It protects macromolecules such as lipids, proteins and DNA from free radical damage through a process of glutathiolation; it acts as a proton donor in the presence of free radicals and plays a role in the production of other antioxidant such as ascorbate.

Secondary metabolites that possess antifungal properties against the genus *Fusarium* [30] that were significantly upregulated in primed versus naïve *S. bicolor* seedlings included shikimic acid, (743 × change at 1 d.p.i. with *F. pseudograminearum*, (Table 2), the precursor of phenylpropanoids that include flavonoids and cinnamate derivatives. Flavonoids that were found to be upregulated included: epicatechin (2 × change at 1 d.p.i.) [31,32,33], hesperetin (3 × change at 1 d.p.i.) [34,35], apigenin (10 × change at 7 d.p.i.) [33,36,37], naringenin (3 × change at 4 d.p.i.) [33,36], kaempferol (6 × change at 4 d.p.i.) [33,36], leucocyanidin (6 × change at 1 d.p.i.) [32] and neohesperidin (2 × change at 4 d.p.i.) [34] (Table 2). Hydroxycinnamic acids and derivatives found to be upregulated included: caffeic acid (5 × change at 7 d.p.i.) [38], coumaroylshikimate [39], caffeoylquinate (7 × change at 1 d.p.i.) [30] and cinnamic acid (2 × change1 d.p.i.) [33,38] (Table 2). 

The group of fatty acids, that were significantly upregulated earlier in primed versus naïve *S. bicolor* seedlings, were γ-linolenic acid (3 × change), octadecenoic acid (7 × change) and oleic acid (446 × change). These fatty acids are found in higher levels in grain varieties that has resistance to *Fusarium*-diseases [40]. Other lipids that were upregulated included sphingosine (288 × change at 4 d.p.i.) and spirilloxanthin (a carotenoid ether, -9 × change at 1 d.p.i.) (Table 2). Sphingosine is a cell membrane fatty acid that plays an important role in signalling and programmed cell death [41,42]. 

The vitamin folic acid was also upregulated in primed plants. Folate, like most vitamins, plays an important role in protecting plants against stress, as it possesses strong antioxidant potential [43,44]. The cyanogenic glucoside dhurrin was significantly upregulated in primed *S. bicolor* seedlings. Cyanogenic glucosides are known to liberate HCN, which is fungitoxic [45]. The occurrence of dhurrin in stems of primed plants infected with *F. pseudograminearum*, indicate that it might have played a role in suppressing *Fusarium* infection [46]. 

Sorghum contains large quantities of phenols and antioxidants, crucial in active defence against biotic [47] and abiotic stresses [48]. In a study done by Tugizimana et al. [47], the defence-related metabolic reprogramming in *S. bicolor* in response to *Colletotrichum sublineolum* inoculation implicated the activation of both the early phenylpropanoid and flavonoid metabolic pathways as part of the defence response. In the current study, similar defence-related metabolites were upregulated in *P. alvei*-primed *S. bicolor* plants in response to *F. pseudograminearum* inoculation during ISR. These results provide evidence for significant overlap in the metabolic reprogramming between non-ISR and ISR mediated defence in *S. bicolor*, including the defence metabolites such as apigenin, naringenin, kaempferol, other flavonoid conjugates and hydroxycinnamate conjugates such as caffeoylquinate. Moreover, when the metabolic defence-related reprogramming of *P. alvei*-primed *S. bicolor* plants is compared to non-primed plants, it is clear that priming resulted in a quicker and/or enhanced upregulation of amino acid-, phytohormone-, flavonoid-, phenylpropanoid- and lipid metabolites in response to inoculation with *F. pseudograminearum* (Figure 5).

## 4. Materials and Methods

### 4.1. Greenhouse Assessment of Induced Systemic Resistance

#### 4.1.1. Inoculum Preparation 

PGPR: A PGPR strain (*Paenibacillus alvei* NAS-6G6) that has previously shown plant growth enhancement and biocontrol activity on cereal crops [49] was obtained from the PGPR collection of the University of Pretoria (Pretoria, South Africa). The bacterial strain was maintained at -72 °C on Microbeads^®^ (Davies Diagnostics, Randburg, South Africa). The strain was streaked onto Nutrient agar and a 1 w old culture was inoculated into Nutrient broth and incubated in a rotary shaker at 25 °C and 150 rpm for 48 h. The bacterial suspension was subsequently centrifuged in 50 mL capacity sterile plastic tubes at 2000 rpm for 10 min. The resulting pellet was re-suspended in quarter strength sterile Ringer’s solution to give a final concentration of 10^8^ cfu mL^−1^. 

Fungal pathogen: *Fusarium pseudograminearum* (strain M7816N) was obtained from Dr. Sandra Lamprecht at the Agricultural Research Council Plant Protection Research Institute, Stellenbosch, South Africa. The strain was maintained in culture on filter paper at 5 °C and, when needed, plated onto half strength potato dextrose agar (PDA) [50]. Five 5 mm diameter discs were subsequently taken from the edge of a 72 h culture and inoculated into 500 mL mungbean liquor medium [51]. 

#### 4.1.2. Sorghum Cultivation

*Sorghum bicolor* seed (cultivar Sweet NS 5655) was obtained from Advance Seed (Krugersdorp, South Africa). The seeds were sterilised successively in 70% ethanol (5 min), 1% sodium hypochlorite (1 min) and rinsed five times with sterile dH_2_O. The seeds were subsequently transferred to Petri dishes containing filter paper moistened with sterile dH_2_O and allowed to germinate for 48 h at 25 °C. The germinating seeds were inspected daily for any bacterial and fungal growth and contaminated seedlings were discarded. The *S. bicolor* germlings were directly planted into plastic seedling trays filled with washed, autoclaved (120 °C for 20 min), pure silica sand. The trays consisted of 30 × 50 mL cells per tray and were sterilised with 10% sodium hypochlorite. The plants were watered every second day with sterilised dH_2_O to field capacity. The seedlings were fertilised once a week with a general water soluble fertiliser (Multifeed^®^, Nulandis, Kempton Park, South Africa). No pesticides or fungicides were needed. The greenhouse temperature was maintained at between 20 °C and 30 °C and the relative humidity fluctuated between 40% and 60%. At harvest, the fresh and dry weights of both roots and shoots were measured and samples for metabolomic analysis were taken as detailed under 4.2.1. The experimental design consisted of three independent biological repeats.

#### 4.1.3. Treatment (Priming) with *Paenibacillus alvei* and Inoculation with *Fusarium pseudograminearum*

PGPR: Seedlings were treated with *P. alvei* 1 d prior to inoculation with *F. pseudograminearum* [52]. The sand around each individual seedling was drenched with 1 mL of the *P. alvei* cell suspension at 10^8^ cfu mL^−1^.

**Figure 6:**
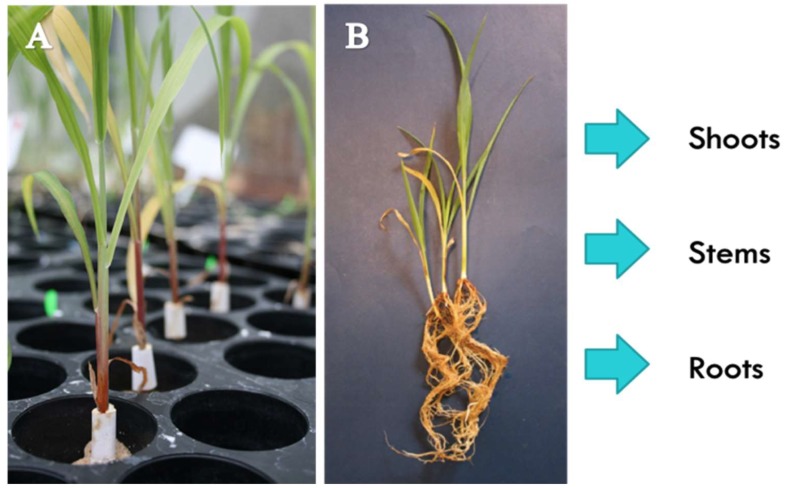
(**A**) Sterilised, absorbent cotton wool wrapped around the stems of *S. bicolor* seedlings to enable pipette-inoculation with a *F. pseudograminearum* spore suspensions. (**B**) Samples were taken from roots (R), stems (S) and shoots (leaves, L) of *F. pseudograminearum* inoculated, *P. alvei* amended, primed (*P. alvei* amended and *F. pseudograminearum* inoculated) and untreated (control) *S. bicolor* seedlings.

Fungal pathogen: One day after treatment of sorghum seedlings with *P. alvei*, the plants were inoculated with the fungal pathogen. To ensure spatial separation from *P. alvei* in the root zone, a small piece (30 × 15 mm) of sterilised absorbent cotton wool was wrapped around the base of each stem as shown in Figure 6, at ca. 1 cm above the surface of the sand and held in place by masking tape (15 mm wide). The plants were pipette-inoculated with the fungal pathogen by adding 500 µL of a conidial suspension of *F. pseudograminearum* onto the cotton wool in a similar fashion as previously described by Mitter et al. 2006 [53]. Final spore suspension concentrations of 1 × 10^2^, 1 × 10^4^ and 1 × 10^6^ spores mL^−1^ were used for inoculation [54,55]. Control treatments received 500 µL of sterile mungbean liquor medium.

#### 4.1.4. Crown Rot Disease Severity and Confirmation of Koch’s Postulates

Li-rating: At harvest, disease severity was assessed at 1, 4, 7 and 14 d.p.i. according to the Wildermuth and McNamara ‘0–5 scale’ as modified by Li et al. [21], where 0 = no obvious symptom; 1 = visible necrotic lesion on coleoptile or first leaf sheath; 2 = the first leaf sheath and below sub-crown internode partially necrotic; 3 = the second leaf sheath and the below sub-crown internode completely necrotic with up to 50% reduction in seedling height; 4 = the third leaf or leaf sheath and the below sub-crown internode partially or completely necrotic with more than 50% reduction of seedling height; 5 = whole plant severely to completely necrotic. 

Fungal isolation: In order to confirm Koch’s postulates, segments from the crown area from both infected and non-infected plants were excised and the surface was sterilised with 0.5% sodium hypochlorite for 2 min and rinsed five times with sterile water. These segments were aseptically plated in triplicate on rose bengal-glycerol-urea (RbGU) medium [20]. The plates were incubated at 27 ± 1 °C for 7 d and the resulting fungal colonies were examined microscopically.

#### 4.1.5. Statistical Analyses of Growth Parameters and Disease Assessments

Data were subjected to analysis of variance and means were compared using Tukey’s least significance determination (LSD) test at a significance level of *p* = 0.05.

### 4.2. Metabolite Profiling

#### 4.2.1. Sample Collection

At harvest, fresh samples were collected from roots, stems and leaves of (1) *F. pseudograminearum*-inoculated, (2) *P. alvei-*amended, (3) primed (*P. alvei* amended and *F. pseudograminearum* inoculated) and (4) untreated (control) *S. bicolor* seedlings (Figure 6). Samples were consecutively taken from each seedling, first from leaves, then stems and lastly roots. Root samples were carefully removed from the growing medium (pure silica sand) by adding sterilised dH_2_O to each pot (up to field capacity) to loosen the roots. The roots were then carefully washed with sterilised dH_2_O and dried with tissue paper. Samples were immediately weighed, placed in 50 mL centrifuge tubes, frozen with liquid nitrogen and kept at −72 °C until time of metabolite extraction. 

Right before extraction, the frozen samples were carefully crushed to a powder by making use of a clean spatula and kept frozen by adding liquid nitrogen as needed. One gram of the crushed sample was then carefully transferred to a sterile 50 mL centrifuge tube. The extraction process followed immediately thereafter making sure that the sample remained frozen up to this point.

#### 4.2.2. Metabolite Extraction

Intracellular metabolites were extracted with 80% methanol [1:10 (w/v)] from roots, stems and leaves of (1) *F. pseudograminearum*-inoculated, (2) *P. alvei*-amended, (3) primed (*P. alvei* amended and *F. pseudograminearum* inoculated) and (4) untreated (control) *S. bicolor* seedlings (Figure 6) at 1, 4 and 7 d.p.i. with *F. pseudograminearum* which coincided with 2-, 5- and 8 d post treatment with *P. alvei,* respectively (it is important to note that the root samples also contained *P. alvei* metabolites and stem samples also contained *F. pseudograminearum* metabolites). The 80% methanol mixture was homogenised using an Ultra Turrax homogeniser. The samples were subsequently centrifuged for 20 min at 5100 rpm at 4°C. Supernatants were removed and evaporated under vacuum by using a rotary evaporator at 55 °C to a final volume of approximately 1 mL and transferred to Eppendorf tubes where it was dried in a vacuum centrifuge at 40 °C for 6 h to complete dryness. The dried samples were subsequently resuspended to a final volume of 500 µL (80% aqueous LC-grade methanol and 20% ultrapure water) and filtered through 0.22 µm nylon syringe filters (Anatech, Randburg, South Africa) into high performance liquid chromatography glass vials fitted with 500 µL inserts and stored at −20 °C. For quality control (QC) purposes, pooled samples were prepared by pipetting and mixing aliquots of equal volume from all samples.

#### 4.2.3. Ultra-High Performance Liquid Chromatography-High Definition Mass Spectrometry Analysis

Methanol extracts were analysed using a Waters Acquity ultra-high performance liquid chromatography coupled in tandem to a Waters SYNAPT G1 quadrupole time-of-flight mass spectrometer (Waters Corporation, Milford, MA, USA). Three technical replicates of each of the four treatments: (1) *F. pseudograminearum* inoculated, (2) *P. alvei* amended, (3) primed (*P. alvei* amended and *F. pseudograminearum* inoculated) and (4) untreated (control) were performed resulting in 12 injections for each of the nine biological groups [plant tissue (roots, stems and leaves) versus time-point (1, 4 and 7 d.p.i.)]. Chromatographic separation of the aqueous-methanol extracts was performed using a Waters HSS T3 C18 column (150 mm × 2.1 mm × 1.8 µm) thermostatted at 60 °C. Although the T3 column is classified as a C18 reverse phase type, it can separate some polar compounds in addition to the non-polar compounds. The elution gradient was carried out with a binary solvent system consisting of 0.1% aqueous formic acid (Sigma-Aldrich, Munich, Germany) (solvent A) and 0.1% formic acid in acetonitrile (Romil Pure Chemistry, Cambridge, UK) (solvent B) at a flow rate of 0.4 mL min^−1^. The initial conditions of 98% A and 2% B were held for 13 min followed by 30% A and 70% B at 14 min. At 15 min, the conditions were changed to 5% A and 95% B; these conditions were held for 2 min and then changed to the initial conditions. The analytical column was allowed to equilibrate for 2 min before the next injection. The total chromatographic run time was 20 min and the injection volume was 2 µL. Each sample was analysed in triplicate to account for any analytical variability. The MS detector acquired data in both positive and negative modes following electrospray ionisation. The conditions were set as follows: capillary voltage of 2.5 kV, sampling cone at 30 V, extraction cone at 4 V, cone gas flow 50 L h^−1^, desolvation gas flow 550 L h^−1^, source temperature at 120 °C, desolvation temperature at 450 °C, scan time of 0.1 s and mass range of 100–1000 Da. Leucine encephalin (50 pg mL^−1^) was used as a calibrant to acquire mass accuracies between 1 and 3 mDa and data were acquired at different collision energies [mass spectrometry at different collision energies (MS^E^) ranging between 10–50 eV] to aid with structural elucidation and annotation of the analytes. Solvent blanks and the QC samples were also analysed in parallel with the sample extracts. The sample acquisition was randomised in the QC sample (six injections) was analysed every 30 injections to monitor and correct changes in the instrument responses. Furthermore, six QC injections were performed in the beginning and end of the batch to insure ensure system equilibration.

#### 4.2.4. Data Analysis

In order to visually assess the data, the sets were processed using MarkerlynxXS™ software (Waters Corporation, Milford, USA). Alignment, peak finding, peak integration and retention time (Rt) correction were done on a Rt range of 1.5 and 15 min, *m/z* range of 100–1000 Da, mass tolerance of 0.05 D and Rt window of 0.2 min. Data were normalised to total intensity (area) using MarkerlynxXS. The datasets thus obtained were exported to the SIMCA (soft independent modelling of class analogy) software version 14 (Umetrics, Umea, Sweden) in order to perform PCA and OPLS-DA. Before performing these multivariate data analyses (MVDA), data were mean centred and Pareto-scaled for both models. The computed and used models were validated as described in the results section. This study mainly focused on the compounds with direct activity against pathogenesis and the associated defence metabolism, thus only the upregulated metabolites were reported on here.

#### 4.2.5. Metabolite Annotation

Metabolites were annotated using Taverna workbench (www.taverna.org.uk) for PUTMEDID_LCMS Metabolite ID Workflows [56,57]. The Taverna workflows allow for integrated, automated and high-throughput annotation and putative metabolite identification from LC-ESI-MS metabolomic data. The workflows consist of correlation analysis, metabolic feature annotation and metabolite annotation. A data matrix from MarkerLynx-based data processing was firstly formatted to match the Taverna workbench requirements. Three main workflows formed the Taverna Metabolite ID procedure: (i) Pearson-based correlation analysis (List_CorrData), (ii) metabolic feature annotation (annotate_Massmatch)—allowing for grouping together ion peaks with similar features such as Rt, and annotating features with the type of *m/z* ion (molecular ion, isotope, adduct, others) believed to originate from the same compound. The elemental composition/molecular formula (MF) of each *m/z* ion was then automatically calculated; and (iii) metabolite annotation (matchMF-MF) of the calculated MF (from the output file from workflow 2) was automatically compared and matched to the MF from a pre-defined reference file of metabolites (inhouse library). 

For confidence in metabolite annotation, the following steps were performed: (i) the calculated MF of a selected metabolite candidate was manually searched against databases and bioinformatics tools, mainly Chemspider (www.chemspider.com) [58], SorghumbicolorCyc (https://www.plantcyc.org/databases/sorghumbicolorcyc/5.0) [59] and KEGG (Kyoto Encyclopedia of Genes and Genomes, www.genome.jp/kegg/) [60] (ii) structural confirmation through careful inspection of fragmentation patterns by examining the MS^1^ and MS^E^ spectra of the selected metabolite candidate; (iii) comparative assessment with/against annotation details of metabolites in *S. bicolor*, reported in literature [61,62]. Metabolites were annotated to level 2 as classified by the Metabolomics Standard Initiative (MSI) [63].

#### 4.2.6. Metabolic Pathway Analysis

Metabolic pathway analysis was performed using the MetPA (Metabolomics Pathway Analysis) component of the MetaboAnalyst bioinformatics tool suite (version 3.0; http://www.metaboanalyst.ca/) [64]. This enabled the visualisation of the affected metabolic pathways for the identified metabolites obtained from the OPLS-DA.

## 5. Conclusions

Priming *S. bicolor* seedlings with *P. alvei* NAS-6G6 resulted in the induction of systemic resistance against *F. pseudograminearum*. Results obtain from an untargeted metabolomics approach using an UHPLC-HDMS analytical platform, indicate that the metabolic reprogramming was attributed to an early, enhanced upregulation of phytohormone-, amino acid-, flavonoid-, phenylpropanoid- and lipid metabolism upon inoculation with *F. pseudograminearum* in primed plants compared with those left naïve. Secondary metabolites/phytoalexins that possess antifungal properties against the genus *Fusarium* were significantly upregulated in *P. alvei*-primed versus naïve *S. bicolor* seedlings. These included epicatechin, hesperetin, coumaroylshikimate, apigenin, naringenin, kaempferol, leucocyanidin, neohesperidin, shikimic acid, caffeic acid, caffeoylquinate and cinnamic acid, giving rise to a significant reduction in crown rot disease severity. These findings were corroborated by the metabolic pathways that were found to be of high significance in the *S. bicolor* response to *P. alvei*-induced priming against *F. pseudograminearum*. These included glutathione metabolism, shikimate/phenylalanine metabolism and flavonoid biosynthesis. This study revealed strong defence-related metabolic reprogramming in primed sorghum seedlings versus naïve plants as early as 1 d.p.i., pointing to the pre-conditioning of the *P. alvei*-primed plants to quickly halt the invasion and establishment of *F. pseudograminearum*.

## Figures and Tables

**Table 2:** Summary of annotated metabolites upregulated in the roots (R), stems (S) and leaves (L) of *F. pseudograminearum* inoculated *S. bicolor* seedlings primed with *P. alvei* versus non-primed (naïve) seedlings. Discriminating metabolites were identified based on OPLS-DA S-plots.

								Metabolite Fold Change (fc) and *p*-Value of Primed *vs.* Naïve Seedlings					
	Metabolite	Heatmap Key	*m/z*	Rt (min)	ESI Mode	Molecular Formula/KEGG ID	Metabolite Class	1 d.p.i.		4 d.p.i.		7 d.p.i.	
								fc	*p*	fc	*p*	fc	*p*
1	N-Carbamoylputrescine	Putrescine R	176.08	4.66	Pos	C_5_H_13_N_3_O/C00436	Amino acid	3.481	1.67 × 10^−5^	5.401	2.00 × 10^−7^	1.426	1.79 × 10^−2^
2	L-2,4-Diaminobutanoate	DABA S	163.05	5.59	Pos	C_4_H_10_N_2_O_2_/C03283	Amino acid	2.373	3.97 × 10^−2^	0.287	4.30 × 10^−2^	1.660	5.44 × 10^−1^
3	THF-L-Glutamate	THF-L-Glu L	595.19	1.65	Neg	C_24_H_30_N_8_O_9_/C09332	Amino acid	2.623	5.39 × 10^−2^	2.770	1.12 × 10^−3^	1.401	3.45 × 10^−1^
4	Glutathione	Glu R	326.12	2.93	Pos	C_10_H_17_N_3_O_6_S/C00051	Amino acid	5.562	4.22 × 10^−2^	0.649	4.74 × 10^−1^	1.045	9.54 × 10^−1^
5	2-(3-Carboxy-3-aminopropyl)-L-histidine	His R	339.07	5.76	Neg	C_10_H_16_N_4_O_4_/C04441	Amino acid	2.845	1.21 × 10^−3^	0.694	7.96 × 10^−2^	0.643	8.00 × 10^−3^
6	2-(3-Carboxy-3-aminopropyl)-L-histidine	His S	301.09	8.89	Pos	C_10_H_16_N_4_O_4_/C04441	Amino acid	2.362	2.08 × 10^−1^	6.096	1.31 × 10^−2^	0.600	3.27 × 10^−1^
7	L-Lysine	Lys R	213.09	2.14	Neg	C_6_H_14_N_2_O_2_/C00047	Amino acid	0.762	4.98 × 10^−1^	4.140	1.05 × 10^−2^	1.256	4.78 × 10^−1^
8	N6,N6,N6-Trimethyl-L-lysine	Trimethyl Lys R	313.09	3.60	Neg	C_9_H_20_N_2_O_2_/C05546	Amino acid	5.498	1.60 × 10^−3^	4.479	1.70 × 10^−4^	4.019	1.2 × 10^−2^
9	L-phenylalanine	Phe R	373.11	5.49	Pos	C_12_H_22_N_4_O_7_/C00079	Amino acid	32.594	3.39 × 10^−2^	20.705	1.92 × 10^−2^	3.180	1.54 × 10^−1^
10	Guanosine 5’-diphosphate	GDP R	675.88	1.15	Neg	C_10_H_17_N_5_O_17_P_4_/C00035	Purine nucleoside	129.214	1.56 × 10^−3^	80.688	1.36 × 10^−4^	48.241	6.52 × 10^−14^
11	8’-Hydroxyabscisate	ABA S	301.11	9.67	Neg	C_15_H_20_O_5_/C15514	Phytohormone	2.403	6.41 × 10^−2^	2.502	7.68 × 10^−3^	0.913	1.98 × 10^−2^
12	Gibberellin A19	GA19 R	429.15	2.85	Neg	C_20_H_26_O_6_/C02034	Phytohormone	2.055	2.94 × 10^−5^	1.777	4.46 × 10^−2^	1.345	3.92 × 10^−2^
13	Gibberellin A8-catabolite	GA8 L	383.11	8.87	Neg	C_19_H_22_O_7_/C11870	Phytohormone	0.819	3.96 × 10^−1^	1.822	4.08 × 10^−6^	1.626	2.11 × 10^−3^
14	Gibberellin A8-catabolite	GA8 R	383.11	8.91	Neg	C_19_H_22_O_7_/C11870	Phytohormone	1.781	1.62 × 10^−8^	1.135	6.42 × 10^−1^	2.585	2.33 × 10^−2^
15	Salicin	Salicin R	353.08	3.65	Neg	C_13_H_18_O_7_/C01451	Phytohormone	14.920	2.96 × 10^−2^	1.278	6.61 × 10^−1^	4.174	1.14 × 10^−1^
16	(-)-11-Hydroxy-9,10-dihydrojasmonic acid 11-beta-D-glucoside	JA R	435.19	9.22	Neg	C_18_H_28_O_9_/C21385	Phytohormone	27.667	1.05 × 10^−3^	78.994	9.42 × 10^−3^	4.373	2.32 × 10^−2^
17	(-)-Jasmonoyl-L-valine	JA-Val R	368.16	5.49	Pos	C_17_H_27_NO_4_/C21509	Phytohormone	25.519	2.37 × 10^−2^	3.025	2.40 × 10^−1^	3.127	2.64 × 10^−1^
18	Dihydrozeatin	Zeatin R	242.10	6.14	Neg	C_10_H_15_N_5_O/C02029	Phytohormone	96.477	2.10 × 10^−3^	15.249	1.08 × 10^−3^	2.199	2.04 × 10^−1^
19	(-)-Epicatechin	Epicatechin S	289.07	3.50	Neg	C_15_H_14_O_6_/C09727	Phenylpropanoid	1.455	7.08 × 10^−3^	2.749	1.50 × 10^−2^	1.299	4.98 × 10^−1^
20	(-)-Hesperetin	Hesperetin S	301.07	8.28	Neg	C_16_H_14_O_6_/C01709	Phenylpropanoid	3.301	1.81 × 10^−2^	3.799	4.39 × 10^−4^	0.697	4.01 × 10^−1^
21	4-Coumaroylshikimate	CoumaroylSA R	336.11	4.70	Neg	C_16_H_16_O_7_/C02947	Phenylpropanoid	4.580	6.11 × 10^−6^	5.575	5.88 × 10^−6^	1.760	5.16 × 10^−6^
22	7-O-D-Glucosyl-apigenin	Apigenin S	477.10	5.95	Neg	C_21_H_20_O_10_/C04608	Phenylpropanoid	0.804	5.84 × 10^−1^	1.935	1.77 × 10^−1^	9.563	3.73 × 10^−4^
23	8-C-Glucosylnaringenin	Naringenin R	433.11	6.00	Neg	C_21_H_22_O_10_/C16492	Phenylpropanoid	0.631	8.01 × 10^−2^	2.588	7.39 × 10^−3^	1.683	6.31 × 10^−2^
24	Kaempferol 3-O-D-Glucosylgalactoside	Kaempferol L	609.15	5.44	Neg	C_27_H_30_O_16_/C16490	Phenylpropanoid	0.877	8.41 × 10^−1^	5.543	3.2 × 10^−3^	1.650	3.54 × 10^−1^
25	Leucocyanidin	Leucocyanidin R	322.09	4.67	Neg	C_15_H_14_O_7_/C05906	Phenylpropanoid	6.203	3.03 × 10^−10^	2.208	6.51 × 10^−3^	1.622	8.90E × 10^−2^
26	Neohesperidin	Neohesperidin R	609.18	4.91	Neg	C_28_H_34_O_15_/C09806	Phenylpropanoid	0.968	6.11 × 10^−1^	2.025	6.52 × 10^−5^	2.579	3.58 × 10^−4^
27	5-O-Caffeoylshikimic acid	Shikimic acid R	352.10	4.01	Neg	C_16_H_16_O_8_/C10434	Phenylpropanoid	743.006	9.33 × 10^−8^	11.814	9.87 × 10^−5^	4.179	4.54 × 10^−5^
28	Caffeic acid 3-glucoside	Caffeic acid R	341.09	6.25	Neg	C_15_H_18_O_9_/C10431	Phenylpropanoid	6.106	5.83 × 10^−2^	6.521	5.20 × 10^−2^	4.845	2.63 × 10^−2^
29	Caffeoylquinate	Caffeoylquinate R	353.09	2.62	Neg	C_16_H_17_KO_9_/C00852	Phenylpropanoid	7.071	2.65 × 10^−2^	4.685	1.35 × 10^−2^	5.660	8.72 × 10^−2^
30	Trans-D-Glucosyl-2-hydroxycinnamate	Cinnamic acid L	325.09	3.71	Neg	C_15_H_18_O_8_/C05158	Phenylpropanoid	1.517	3.37 × 10^−1^	3.486	4.97 × 10^−2^	1.485	3.20 × 10^−1^
31	Trans-D-Glucosyl-2-hydroxycinnamate	Cinnamic acid R	325.09	3.75	Neg	C_15_H_18_O_8_/C05158	Phenylpropanoid	1.907	1.33 × 10^−1^	2.338	2.18 × 10^−2^	1.224	4.75 × 10^−1^
32	Trans-D-Glucosyl-2-hydroxycinnamate	Cinnamic acid S	325.09	6.94	Neg	C_15_H_18_O_8_/C05158	Phenylpropanoid	1.644	9.05 × 10^−3^	2.238	4.44 × 10^−5^	1.634	1.73 × 10^−1^
33	(6Z,9Z,12Z)-Octadecatrienoic acid	γ-linolenic acid R	345.20	1.92	Neg	C_18_H_30_O_2_/C06426	Lipid	3.122	1.18 × 10^−8^	3.157	4.37 × 10^−8^	2.392	2.61 × 10^−9^
34	9-Hydroperoxy-12,13-epoxy-10-octadecenoic acid	OA L	371.18	5.27	Neg	C_18_H_32_O_4_/C08368	Lipid	445.872	2.44 × 10^−14^	691.790	2.44 × 10^−14^	818.658	2.44 × 10^−14^
35	12,13-Epoxy-9-hydroxy-10-octadecenoate	Oleic acid R	361.20	1.14	Neg	C_18_H_32_O_4_/C14832	Lipid	2.041	2.15 × 10^−8^	1.459	1.17 × 10^−5^	1.322	1.04 × 10^−3^
36	Methyl 9-hydroperoxy-10,12,13,15-bisepidioxy-16E-octadecenoate	Oleic acid L	461.14	1.60	Neg	C_19_H_32_O_8/_C14832	Lipid	3.756	4.42 × 10^−3^	0.419	2.13 × 10^−1^	0.968	9.51 × 10^−1^
37	18-Fluoro-octadecanoic acid	Fluoro-OA R	347.23	1.57	Pos	C_18_H_35_FO_2_/C01530	Lipid	76.144	1.20 × 10^−2^	176.354	7.82 × 10^−9^	474.046	6.61 × 10^−4^
38	(4E,8E,10E-d18:3) Sphingosine	Sphingosine R	318.24	1.02	Pos	C_18_H_33_NO_2_/C00319	Lipid	9.621	2.96 × 10^−1^	288.427	2.68 × 10^−2^	111.911	2.14 × 10^−1^
39	3,4,3′,4′-tetrahydrospirilloxanthin	Spirilloxanthin R	673.42	1.21	Neg	C_42_H_64_O_2_/C15888	Lipid	9.271	2.34 × 10^−2^	3.857	5.20 × 10^−3^	1.329	9.69 × 10^−2^
40	5-Formiminotetrahydrofolate	Formimino-THF L	516.14	5.12	Neg	C_20_H_24_N_8_O_6_/C00664	Vitamin	3.140	3.34 × 10^−3^	1.363	3.79 × 10^−1^	2.074	6.33 × 10^−2^
41	Dhurrin	Dhurrin S	310.09	2.82	Neg	C_14_H_17_NO_7_/C05143	Cyanogenic glucoside	2.186	5.67 × 10^−4^	4.514	1.96 × 10^−3^	2.545	1.04 × 10^−1^

## Data Availability

The study design information, LC-MS raw data, analyses and data processing information, and the meta-data are being deposited to the EMBL-EBI metabolomics repository—MetaboLights50, with the identifier MTBLS1111 (http://www.ebi.ac.uk/metabolights/MTBLS1111).

## Supplementary Materials

The supplementary material for this article can be found online at https://www.mdpi.com/2218-1989/9/7/150/s1, Figure S1. (A) Microscopic identification of *F. pseudograminearum* at 400 × magnification. (B) Conidial morphology of *F. pseudograminearum* taken from Aoki et al. [65]. Figure S2. UHPLC-HDMS BPI chromatograms of ESI-positive data indicating the metabolomic profiles of untreated (black), naïve infected (blue) and primed infected (green) stems obtained at 1 d.p.i. with *F. pseudograminearum*. Figure S3. UHPLC-HDMS BPI chromatograms of ESI-positive data indicating the metabolomic profiles of untreated (black), naïve infected (blue) and primed infected (green) leaves obtained at 1 d.p.i. with *F. pseudograminearum*. Figure S4. PCA score/scatter plot of stem samples computed from ESI-positive data. Figure S5. PCA score/scatter plot of leaf samples computed from ESI-positive data. Figure S6. PCA score/scatter plot of root samples computed from ESI-negative data. Figure S7. PCA score/scatter plot of stems samples computed from ESI-negative data. Figure S8. PCA score/scatter plot of leaves samples computed from ESI-negative data. Figure S9. OPLS-DA modelling and variable/feature selection ESI-positive data (stem samples). Figure S10. OPLS-DA modelling and variable/feature selection ESI-positive data (leaf samples). Table S1. Summary of the description and validation of all the generated OPLS-DA models separating naïve versus primed *S. bicolor* plants. Figure S11. Summary of pathway analysis with MetPA. Figure S12. Venn diagram comparing the number of metabolites shown in Table 2 that were significantly upregulated at 1 d.p.i. (blue), 4 d.p.i. (yellow) and 7 d.p.i. (green) with *F. pseudograminearum* in primed versus naïve *S. bicolor* seedlings.
