# Supplementary material for: Differential Metabolic Reprogramming in Paenibacillus alvei-Primed Sorghum bicolor Seedlings in Response to Fusarium pseudograminearum Infection
Source: Metabolites. 2019 Jul 23;9(7):150. doi: 10.3390/metabo9070150 (PMC6680708; doi:10.3390/metabo9070150)
Supplement: Supplementary file 1 [file metabolites-09-00150-s001.zip › Supplementary material/Supplementary material.docx]

*Research article – Supplementary data*

Differential Metabolic Reprogramming in *Paenibacillus* *alvei*-Primed *Sorghum* *bicolor* Seedlings in Response to *Fusarium pseudograminearum* Infection

René Carlson ^1^, Fidele Tugizimana ^2^, Paul A. Steenkamp ^2^, Ian A. Dubery ^2^ and Nico Labuschagne ^1,^*

^1^ Department of Plant and Soil Sciences, Faculty of Plant Pathology, University of Pretoria, Private Bag X20, Hatfield, Pretoria, 0028, South Africa

^2^ Centre for Plant Metabolomics Research, Department of Biochemistry, Faculty of Science, University of Johannesburg, P.O. Box 524, Auckland Park, Johannesburg, South Africa

* Correspondence: [nico.labuschagne@up.ac.za](mailto:nico.labuschagne@up.ac.za); Tel.: +27-827-807-849

Received: 25 April 2019; Accepted: 10 July 2019; Published: date

**Supplementary materials**

Considering the enormous scale of the raw data in addition to the extent of the treatment interactions [Treatment (4) × Time-interval (3) × Tissue (3) × ESI-charge (2)], not all of the chemometric models were included here, but can be made available upon request.


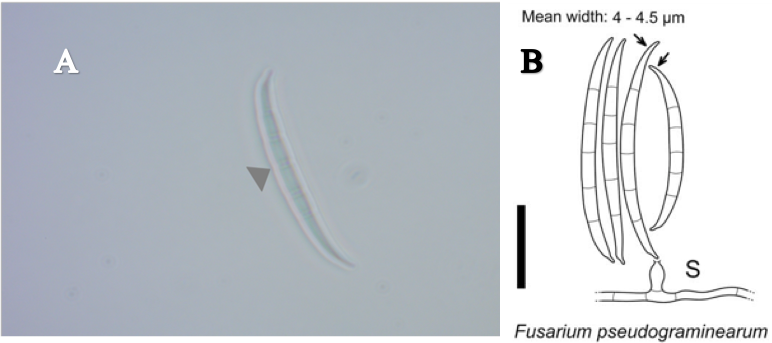


**Figure S1.** (**A**) Microscopic identification of *Fusarium pseudograminearum* at 400× magnification. The grey arrow indicates the widest position of the sporodochial conidium. (**B**) Conidial morphology of *F. pseudograminearum* taken from Aoki *et al.* [59].

**Figure S2.** UHPLC-HDMS BPI chromatograms of ESI-positive data indicating the metabolomic profiles of untreated (black), naïve infected (blue) and primed infected (green) stems obtained at 1 d.p.i. with *F. pseudograminearum*.

**Figure S3.** UHPLC-HDMS BPI chromatograms of ESI-positive data indicating the metabolomic profiles of untreated (black), naïve infected (blue) and primed infected (green) leaves obtained at 1 d.p.i. with *F. pseudograminearum.*

**
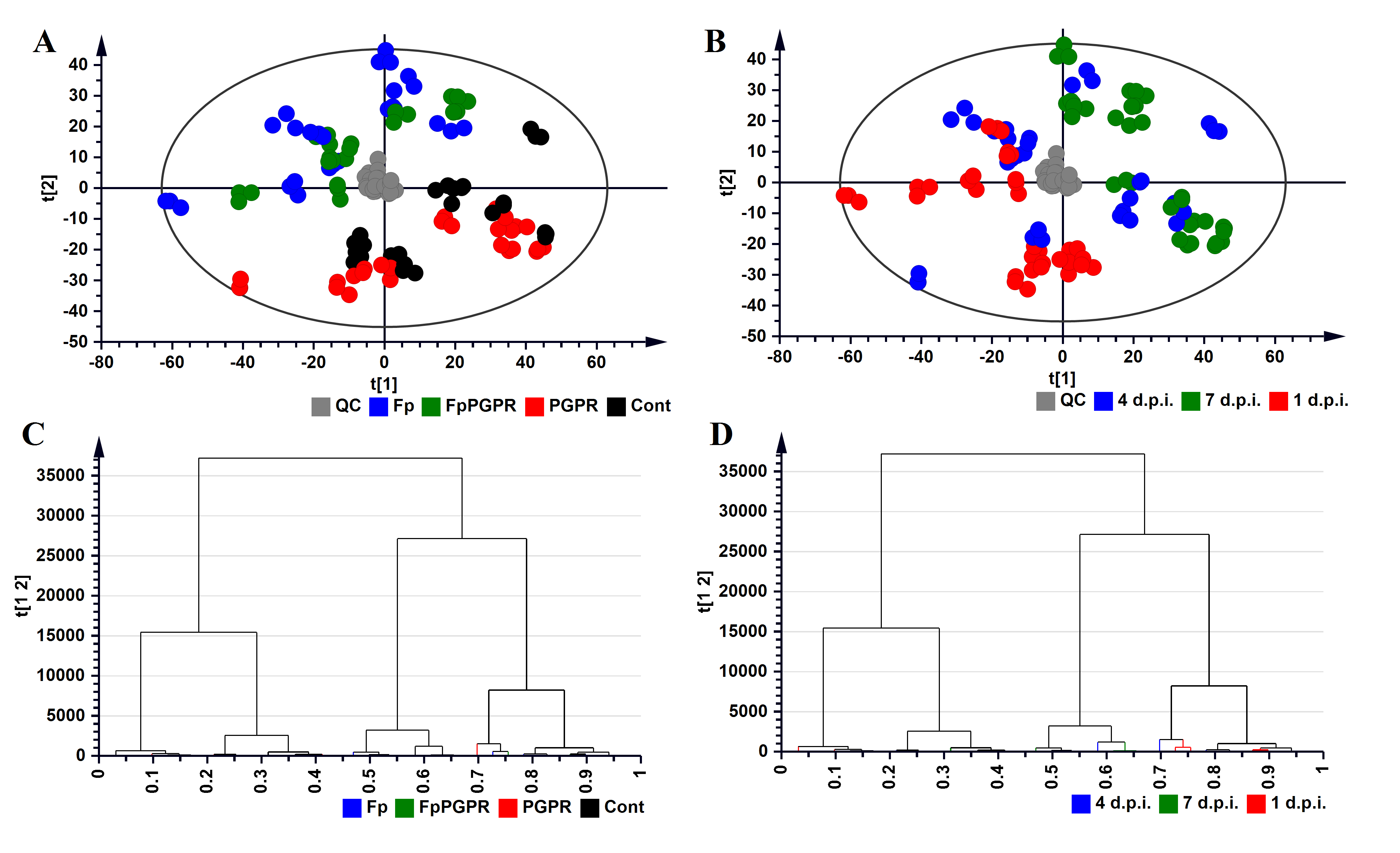
**

**Figure S4.** PCA score / scatter plot of stem samples computed from ESI-positive data representing the first two PCs of an 18-component PCA model. The model explains 81.5% variation in the Pareto-scaled data (R^2^X = 81.5) and 61.8% predicted variation according to cross-validation (Q^2^ =0.618). (**A**,**B**) represents the same PCA scores plot with (**A**) showing the treatment-related clustering and (**B**) showing the time-related clustering. (**C**,**D**) HCA dendrogram corresponding to (**A**,**B**). Legend: QC: Quality control (grey); Fp: Naïve plants inoculated with *F. pseudograminearum* (blue); FpPGPR: *Paenibacillus alvei*-primed plants inoculated with *F. pseudograminearum* (green); PGPR: *P. alvei*-primed plants (red); Cont: Untreated plants (black); 1 d.p.i.: 1 d.p.i. with *F. pseudograminearum* (red); 4 d.p.i.: 4 d.p.i. with *F. pseudograminearum* (blue); 7 d.p.i.: 7 d.p.i. with *F. pseudograminearum* (green).

**
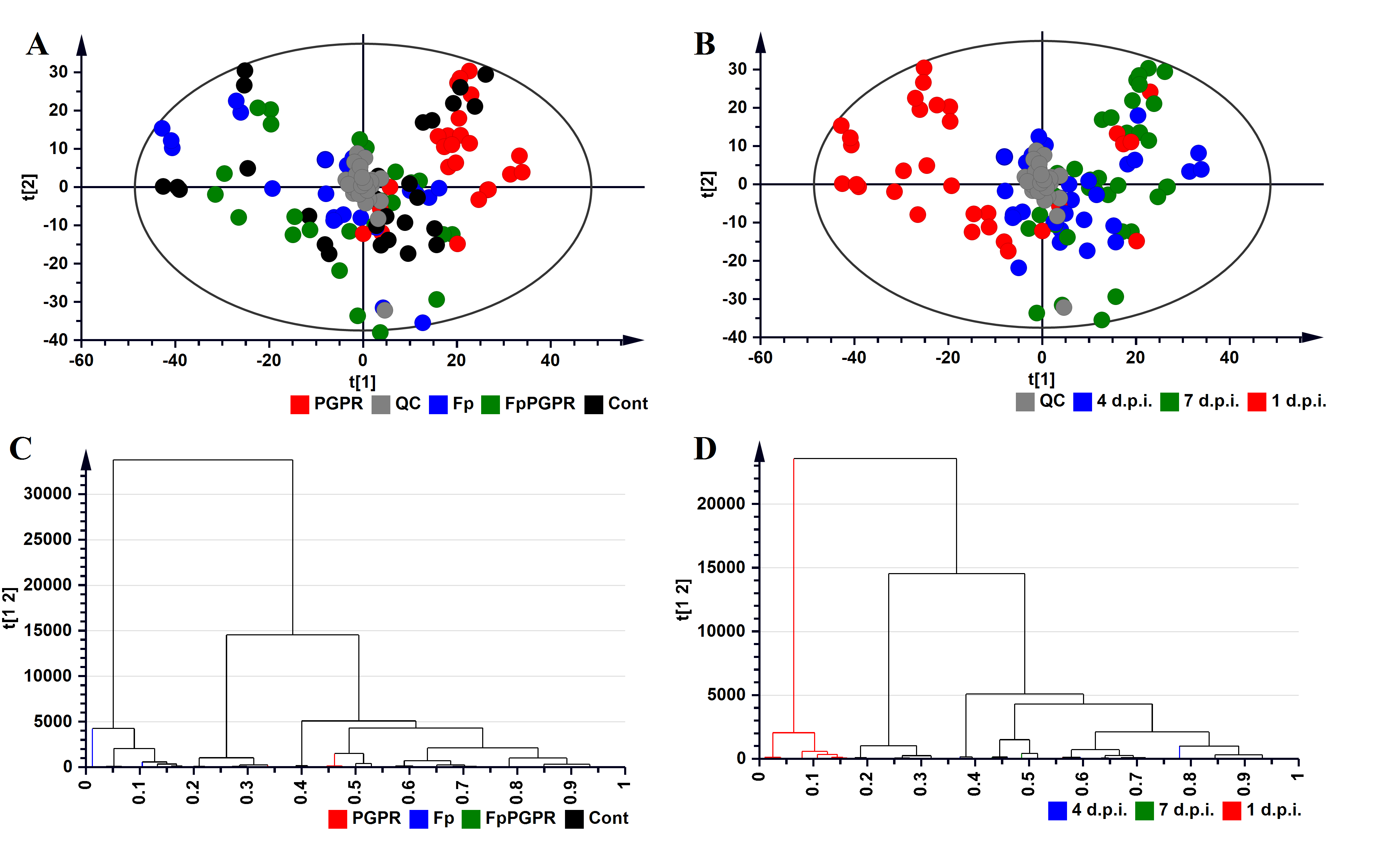
**

**Figure S5.** PCA score / scatter plot of leaf samples computed from ESI-positive data representing the first two PCs of an 16-component PCA model. The model explains 67.5% variation in the Pareto-scaled data (R^2^X = 0.675) and 37.6% predicted variation according to cross-validation (Q^2^ =0.376). (**A**,**B**) represents the same PCA scores plot with (**A**) showing the treatment-related clustering and (**B**) showing the time-related clustering. (**C**,**D**) HCA dendrogram corresponding to (**A**,**B**). Legend: QC: Quality control (grey); Fp: Naïve plants inoculated with *F. pseudograminearum* (blue); FpPGPR: *P. alvei*-primed plants inoculated with *F. pseudograminearum* (green); PGPR: *P. alvei*-primed plants (red); Cont: Untreated plants (black); 1 d.p.i.: 1 d.p.i. with *F. pseudograminearum* (red); 4 d.p.i.: 4 d.p.i. with *F. pseudograminearum* (blue); 7 d.p.i.: 7 d.p.i. with *F. pseudograminearum* (green).


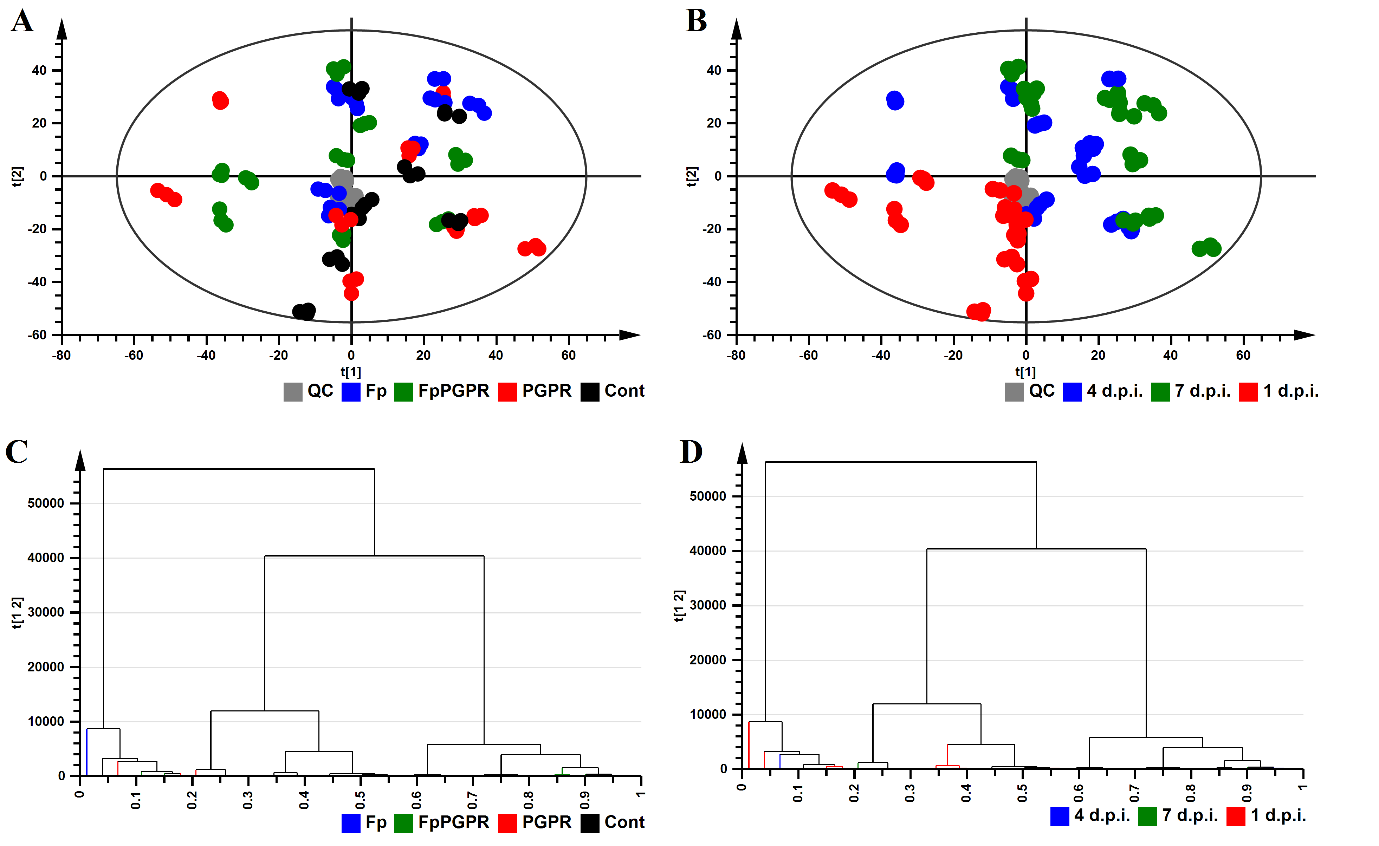


**Figure S6.** PCA score / scatter plot of root samples computed from ESI-negative data representing the first two PCs of a 15-component PCA model. The model explains 66.4% variation in the Pareto-scaled data (R^2^X = 0.664) and 48.6% predicted variation according to cross-validation (Q^2^ =0.486). (**A** and **B**) represents the same PCA scores plot with (**A**) showing the treatment-related clustering and (**B**) showing the time-related clustering. (**C** and **D**) HCA dendrograms corresponding to (**A** and **B**). Legend: QC: Quality control (grey); Fp: Naïve plants inoculated with *F. pseudograminearum* (blue); FpPGPR: *P. alvei*-primed plants inoculated with *F. pseudograminearum* (green); PGPR: *P. alvei*-primed plants (red); Cont: Untreated plants (black); 1 d.p.i.: 1 d.p.i. with *F. pseudograminearum* (red); 4 d.p.i.: 4 d.p.i. with *F. pseudograminearum* (blue); 7 d.p.i.: 7 d.p.i. with *F. pseudograminearum* (green).


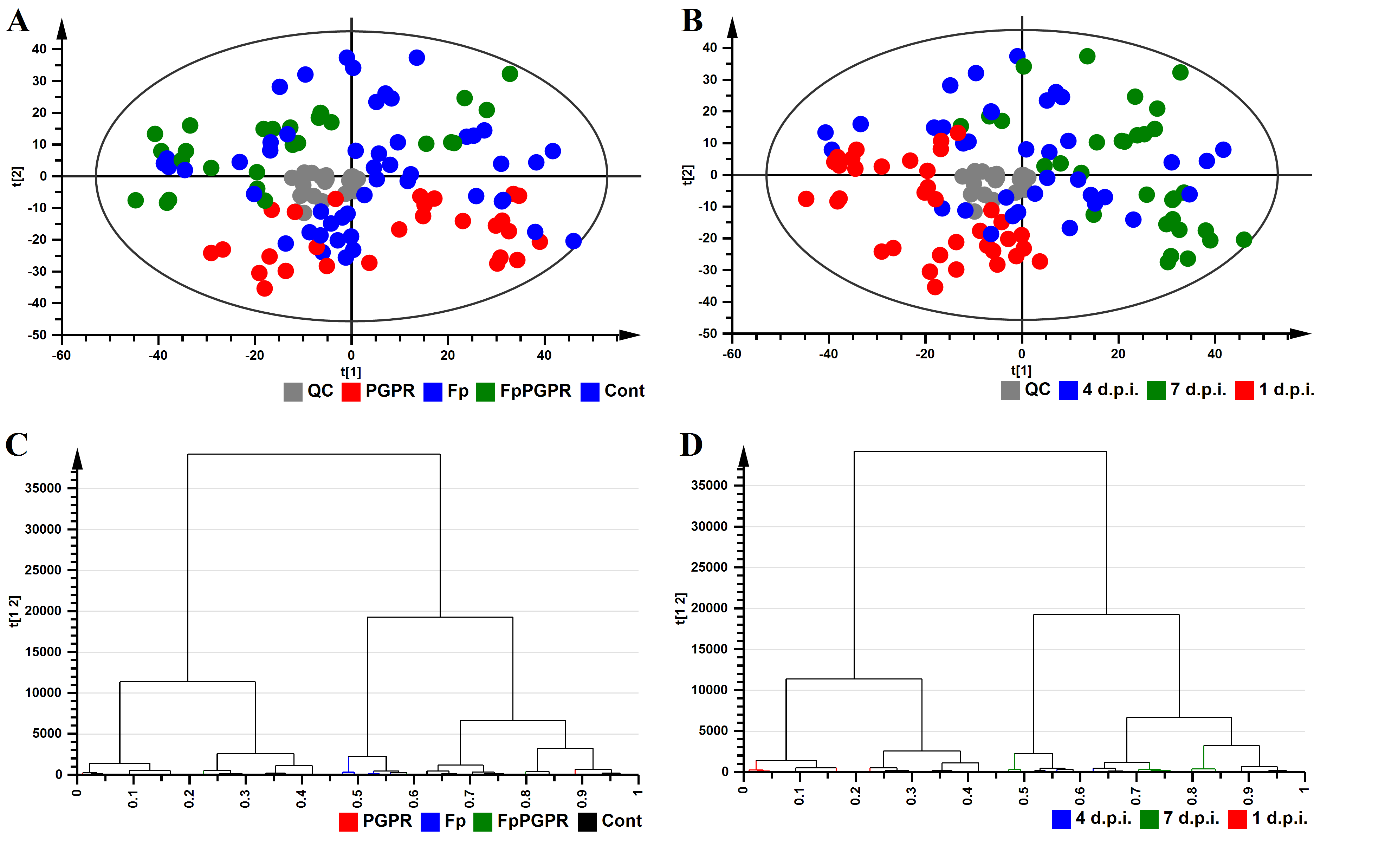


**Figure S7.** PCA score / scatter plot of stems samples computed from ESI-negative data representing the first two PCs of a 15-component PCA model. The model explains 63.8% variation in the Pareto-scaled data (R^2^X = 0.638) and 31.1% predicted variation according to cross-validation (Q^2^ =0.311). (**A** and **B**) represents the same PCA scores plot with (**A**) showing the treatment-related clustering and (**B**) showing the time-related clustering. (**C** and **D**) HCA dendrograms corresponding to (**A** and **B**). Legend: QC: Quality control (grey); Fp: Naïve plants inoculated with *F. pseudograminearum* (blue); FpPGPR: *P. alvei*-primed plants inoculated with *F. pseudograminearum* (green); PGPR: *P. alvei*-primed plants (red); Cont: Untreated plants (black); 1 d.p.i.: 1 d.p.i. with *F. pseudograminearum* (red); 4 d.p.i.: 4 d.p.i. with *F. pseudograminearum* (blue); 7 d.p.i.: 7 d.p.i. with *F. pseudograminearum* (green).


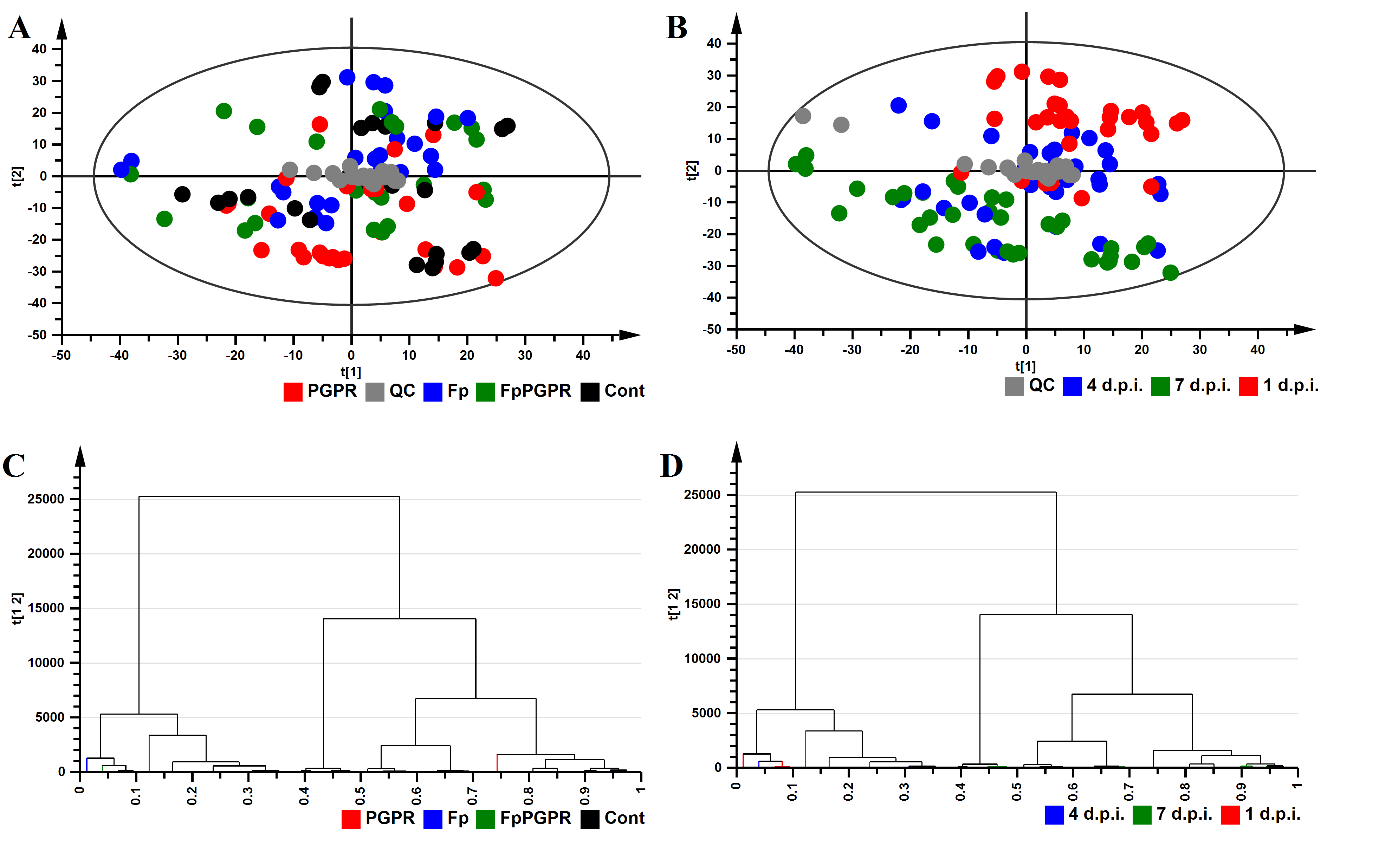


**Figure S8.** PCA score / scatter plot of leaves samples computed from ESI-negative data representing the first two PCs of a 13-component PCA model. The model explains 57.8% variation in the Pareto-scaled data (R^2^X = 0.578) and 33.8% predicted variation according to cross-validation (Q^2^ =0.338). (**A** and **B**) represents the same PCA scores plot with (**A**) showing the treatment-related clustering and (**B**) showing the time-related clustering. (**C** and **D**) HCA dendrograms corresponding to (**A** and **B**). Legend: QC: Quality control (grey); Fp: Naïve plants inoculated with *F. pseudograminearum* (blue); FpPGPR: *P. alvei*-primed plants inoculated with *F. pseudograminearum* (green); PGPR: *P. alvei*-primed plants (red); Cont: Untreated plants (black); 1 d.p.i.: 1 d.p.i. with *F. pseudograminearum* (red); 4 d.p.i.: 4 d.p.i. with *F. pseudograminearum* (blue); 7 d.p.i.: 7 d.p.i. with *F. pseudograminearum* (green).


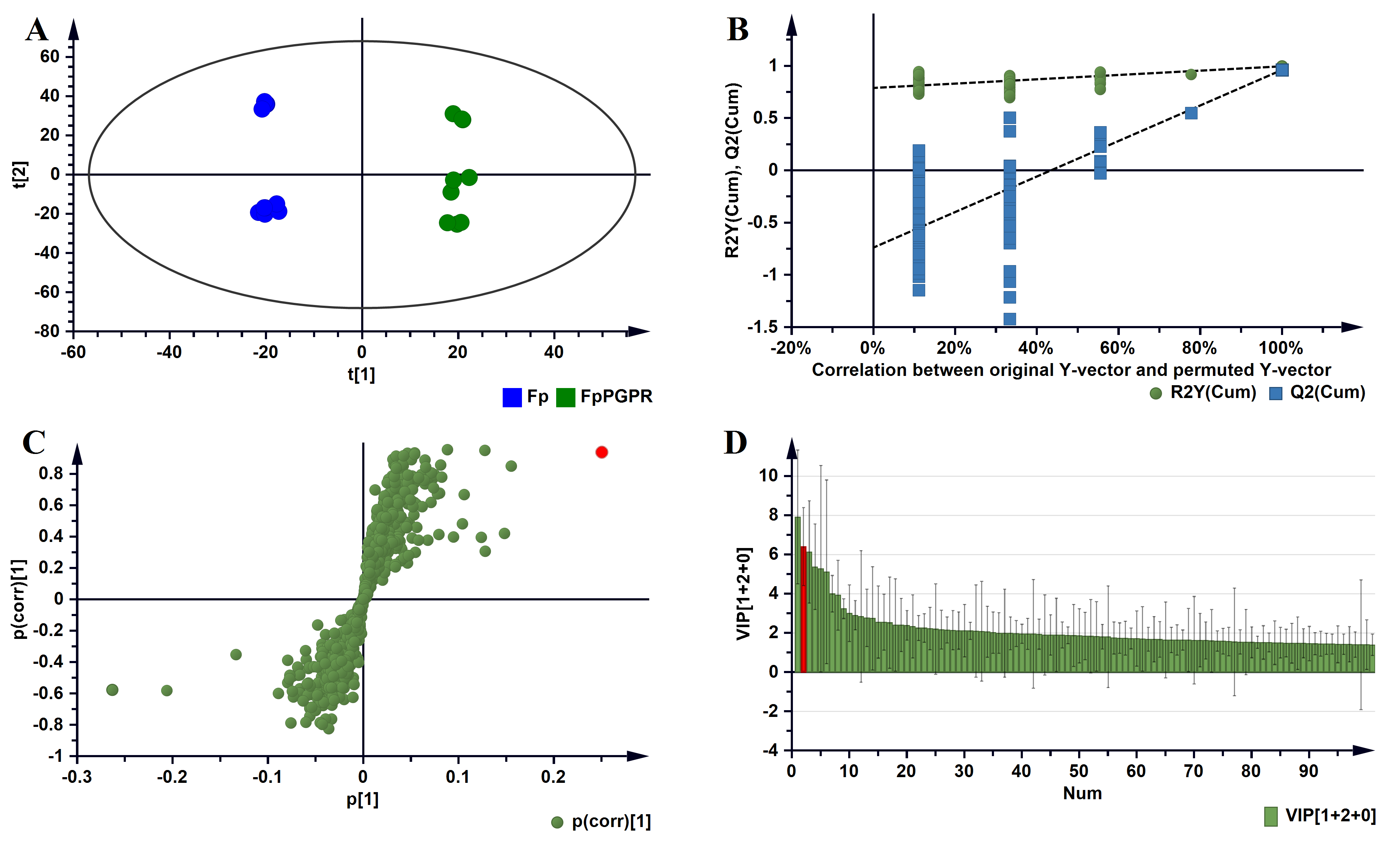


**Figure S9.** OPLS-DA modelling and variable/feature selection ESI-positive data (stem samples). (**A**) A typical PCA scores / scatter plot for the OPLS-DA model separating naïve (Fp) vs primed plants (FpPGPR) at 1 d.p.i. (1 + 2 + 0 components, R^2^X = 0.615, Q^2^ = 0.961, CV-ANOVA *p*-value < 0.05). In the scores plot, it is evident that the two groups are clearly separated: naïve vs primed. (**B**) A typical response permutation test plot (n = 100) for the OPLS-DA model in (A); the R^2^ and Q^2^ values of the permutated models correspond to *y*-axis intercepts: R^2^ = (0.0, 0.807) and Q^2^ = (0.0, -0.516). (**C**) An OPLS-DA loadings S-plot for the same model in (A); variables situated in the extreme end of the S-plot are statistically relevant and represent prime candidates as discriminating variables/features. (**D**) A variable importance for the projection (VIP) plot for the same model; pointing mathematically to the importance of each variable (feature) in contributing to group separation in the OPLS-DA model. (C,D) Examples of the variables that were significantly up-regulated in primed plants are highlighted in red.


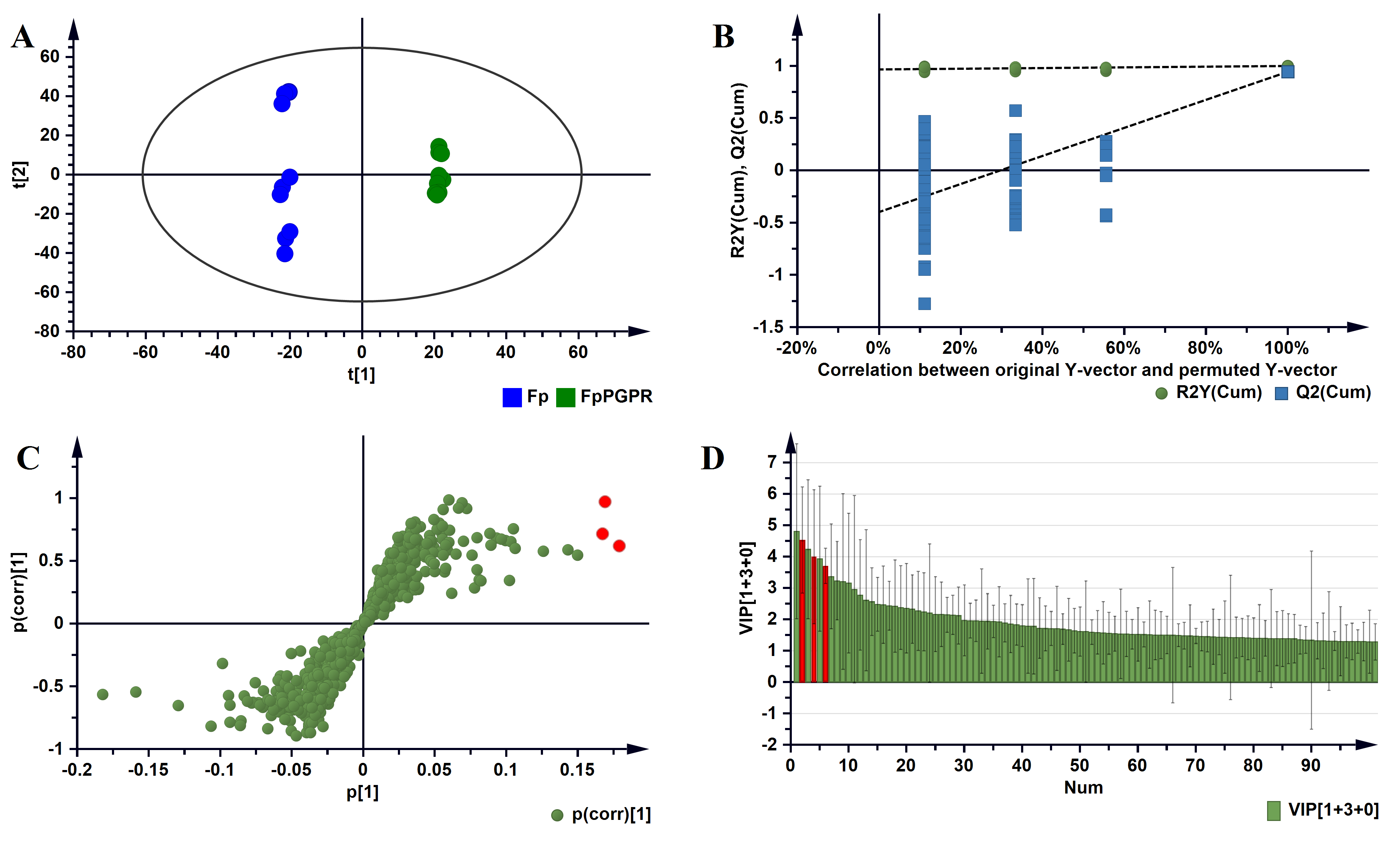


**Figure S10.** OPLS-DA modelling and variable/feature selection ESI-positive data (leaf samples). (**A**) A typical PCA scores / scatter plot for the OPLS-DA model separating naïve (Fp) vs primed plants (FpPGPR) at 1 d.p.i. (1 + 3 + 0 components, R^2^X = 0.594, Q^2^ = 0.943, CV-ANOVA *p*-value < 0.05). In the scores plot, it is evident that the two groups are clearly separated: naïve vs primed. (**B**) A typical response permutation test plot (n = 100) for the OPLS-DA model in (A); the R^2^ and Q^2^ values of the permutated models correspond to *y*-axis intercepts: R^2^ = (0.0, 0.974) and Q^2^ = (0.0, -0.435). (**C**) An OPLS-DA loadings S-plot for the same model in (A); variables situated in the extreme end of the S-plot are statistically relevant and represent prime candidates as discriminating variables/features. (**D**) A variable importance for the projection (VIP) plot for the same model; pointing mathematically to the importance of each variable (feature) in contributing to group separation in the OPLS-DA model. (C,D) Examples of the variables that were significantly up-regulated in primed plants are highlighted in red.

**Table S1.** Summary of the description and validation of all the generated OPLS-DA models separating naïve *versus* primed *S. bicolor* plants.

|  |  |  |  |  |  |  |  |  |  |
| --- | --- | --- | --- | --- | --- | --- | --- | --- | --- |
|  |  |  | **Principle Component Analysis** | | | |  | **Response permutation**^b^ | |
| **Ionisation mode** | **Tissue** | **d.p.i.**^a^ | **Components** | **R^2^X** | **Q^2^** | ***p*-value** |  | **R^2^** | **Q^2^** |
| Neg | Leaves | 1 | 1 + 6 + 0 | 0.705 | 0.906 | < 0.05 |  | (0.0; 1) | (0.0; 0.0) |
| Neg | Leaves | 4 | 1 + 4 + 0 | 0.506 | 0.949 | < 0.05 |  | (0.0; 1) | (0.0; -0.3) |
| Neg | Leaves | 7 | 1 + 4 + 0 | 0.532 | 0.854 | < 0.05 |  | (0.0; 1) | (0.0; -0.1) |
| Pos | Leaves | 1 | 1 + 3 + 0 | 0.594 | 0.943 | < 0.05 |  | (0.0; 1) | (0.0; -0.4) |
| Pos | Leaves | 4 | 1 + 3 + 0 | 0.529 | 0.952 | < 0.05 |  | (0.0; 1) | (0.0; -0.3) |
| Pos | Leaves | 7 | 1 + 4 + 0 | 0.533 | 0.907 | < 0.05 |  | (0.0; 1) | (0.0; -0.2) |
| Neg | Roots | 1 | 1 + 3 + 0 | 0.73 | 0.979 | < 0.05 |  | (0.0; 1) | (0.0; -0.4) |
| Neg | Roots | 4 | 1 + 3 + 0 | 0.639 | 0.960 | < 0.05 |  | (0.0; 1) | (0.0; -0.4) |
| Neg | Roots | 7 | 1 + 6 + 0 | 0.721 | 0.944 | < 0.05 |  | (0.0; 1) | (0.0; -0.2) |
| Pos | Roots | 1 | 1 + 2 + 0 | 0.630 | 0.980 | < 0.05 |  | (0.0; 0.9) | (0.0; -0.5) |
| Pos | Roots | 4 | 1 + 2 + 0 | 0.541 | 0.957 | < 0.05 |  | (0.0; 1) | (0.0; -0.4) |
| Pos | Roots | 7 | 1 + 2 + 0 | 0.514 | 0.967 | < 0.05 |  | (0.0; 1) | (0.0; -0.4) |
| Neg | Stems | 1 | 1 + 6 + 0 | 0.655 | 0.916 | < 0.05 |  | (0.0; 1) | (0.0; 0.0) |
| Neg | Stems | 4 | 1 + 3 + 0 | 0.562 | 0.899 | < 0.05 |  | (0.0; 1) | (0.0; -0.3) |
| Neg | Stems | 7 | 1 + 3 + 0 | 0.577 | 0.879 | < 0.05 |  | (0.0; 1) | (0.0; -0.4) |
| Pos | Stems | 1 | 1 + 2 + 0 | 0.615 | 0.961 | < 0.05 |  | (0.0; 1) | (0.0; -0.6) |
| Pos | Stems | 4 | 1 + 3 + 0 | 0.765 | 0.951 | < 0.05 |  | (0.0; 1) | (0.0; -0.5) |
| Pos | Stems | 7 | 1 + 3 + 0 | 0.730 | 0.974 | < 0.05 |  | (0.0; 1) | (0.0; -0.5) |

^a^ d.p.i.: Days post inoculation with *F. pseudograminearum*.

^b^ Permutated models (n = 100) for each of the OPLS-DA models.


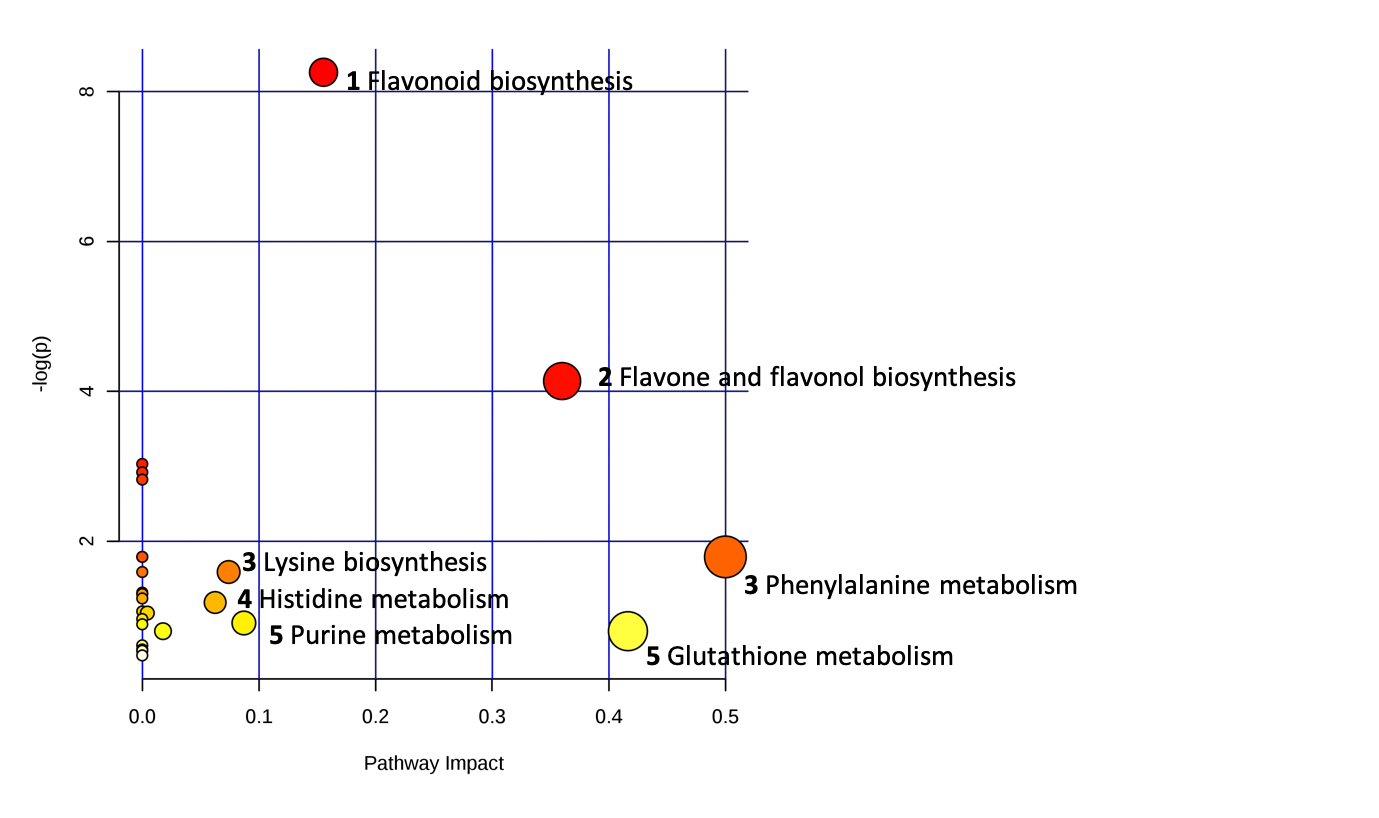


**Figure S11.** Summary of pathway analysis with MetPA: Representation of all MetPA-computed metabolic pathways displayed per their significance or pathway impact. The graph, or “metabolome view” contains all the matched pathways (the metabolome) arranged by *p* values (pathway enrichment analysis) on y-axis, and the pathway impact values (pathway topology analysis) on x-axis. The node color is based on the *p* value and the node radius is defined by the pathway impact values. The latter is the cumulative percentage from the matched metabolite nodes. Thus, the graph indicates pathways with high impact: e.g. flavonoid, flavone and flavonol biosynthesis and phenylalanine and glutathione metabolism to be highly significant metabolic pathways that are involved in the *S. bicolor* response to *P. alvei*-induced priming against *F. pseudograminearum*.


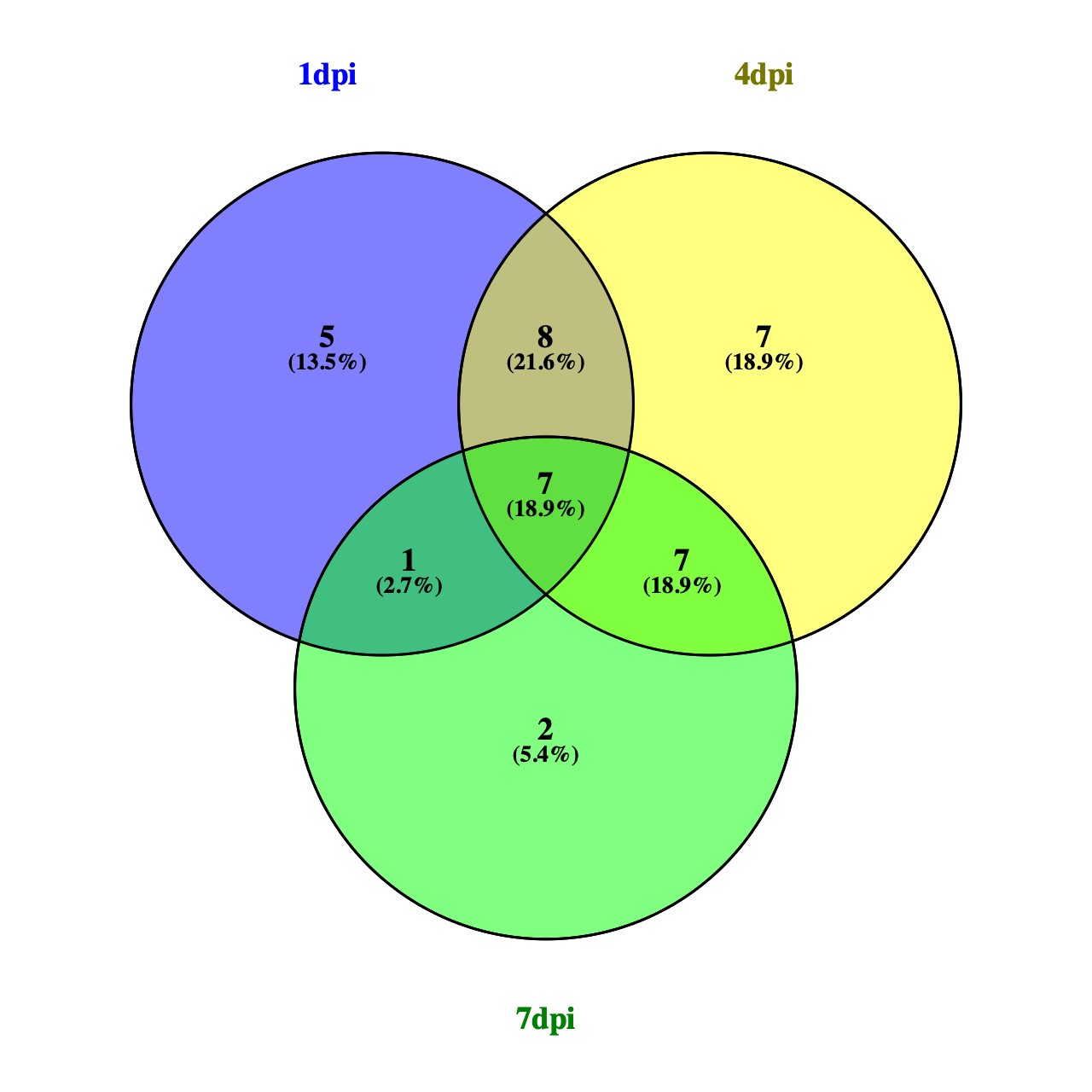


**Figure S12.** Venn diagram comparing the number of metabolites shown in Table 2 that were significantly upregulated at 1 d.p.i. (blue), 4 d.p.i. (yellow) and 7 d.p.i. (green) with *F. pseudograminearum* in primed *versus* naïve *S. bicolor* seedlings.
