# Supplementary figures and images for: Differential Metabolic Reprogramming in Paenibacillus alvei-Primed Sorghum bicolor Seedlings in Response to Fusarium pseudograminearum Infection
Source: Metabolites. 2019 Jul 23;9(7):150. doi: 10.3390/metabo9070150 (PMC6680708; doi:10.3390/metabo9070150)

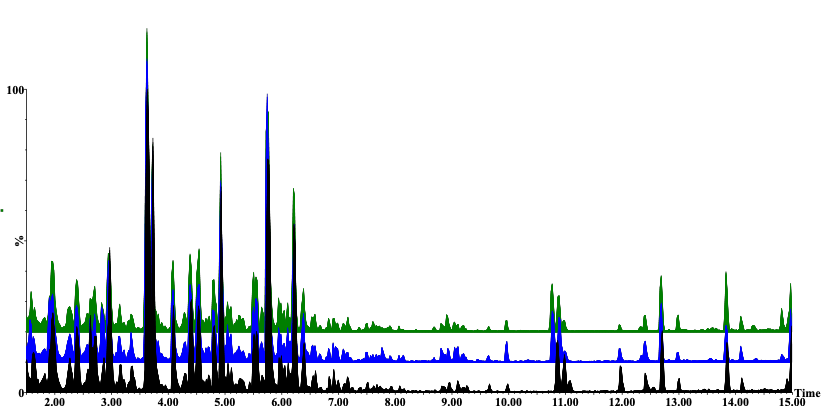

Supplement: Supplementary file 1 [file metabolites-09-00150-s001.zip › Supplementary material/Figure S3.tiff]

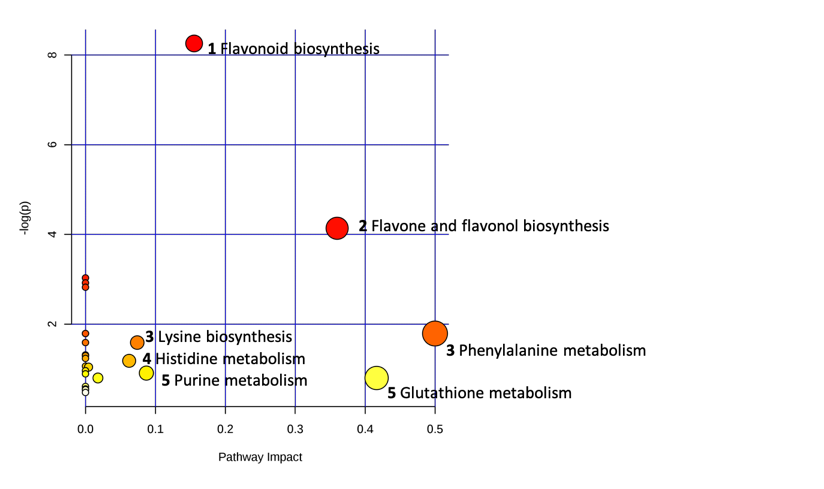

Supplement: Supplementary file 1 [file metabolites-09-00150-s001.zip › Supplementary material/Figure S11.tiff]

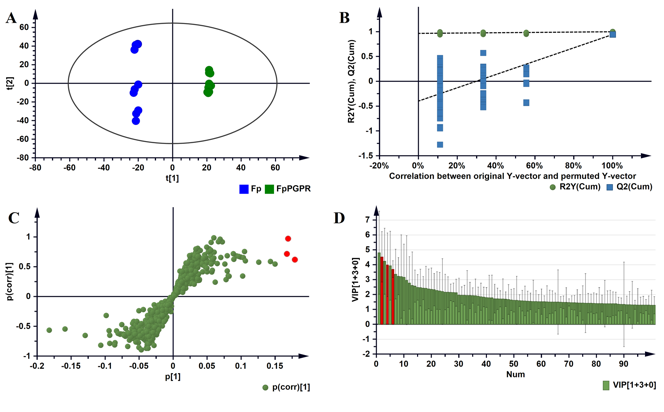

Supplement: Supplementary file 1 [file metabolites-09-00150-s001.zip › Supplementary material/Figure S10.tiff]

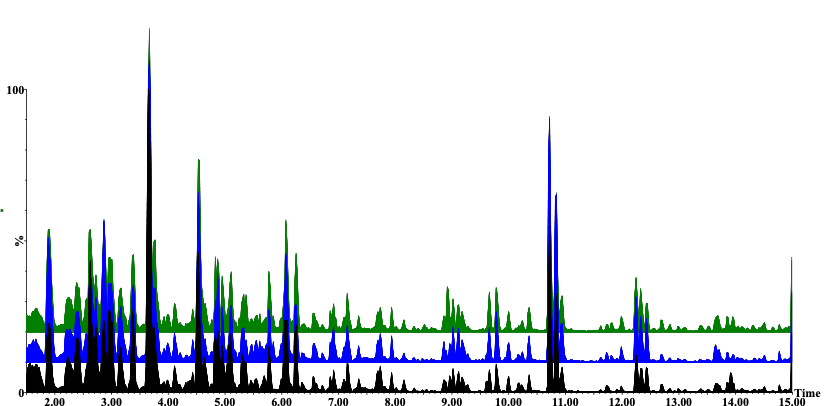

Supplement: Supplementary file 1 [file metabolites-09-00150-s001.zip › Supplementary material/Figure S2.tiff]

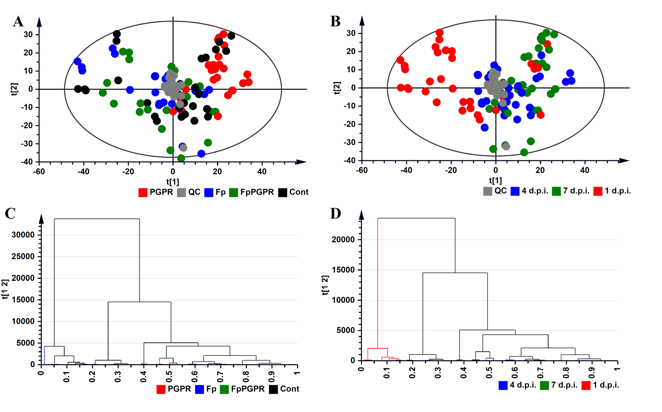

Supplement: Supplementary file 1 [file metabolites-09-00150-s001.zip › Supplementary material/Figure S5.tiff]

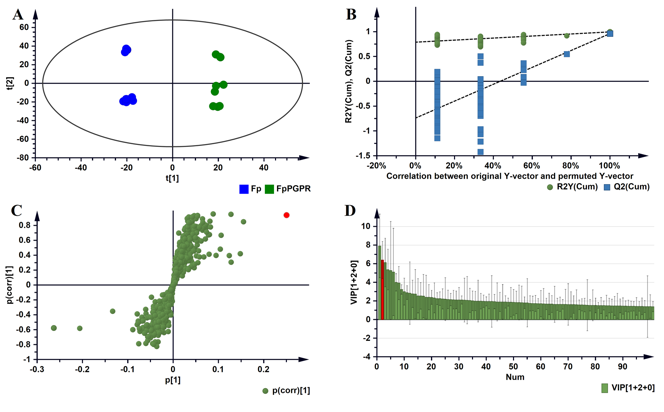

Supplement: Supplementary file 1 [file metabolites-09-00150-s001.zip › Supplementary material/Figure S9.tiff]

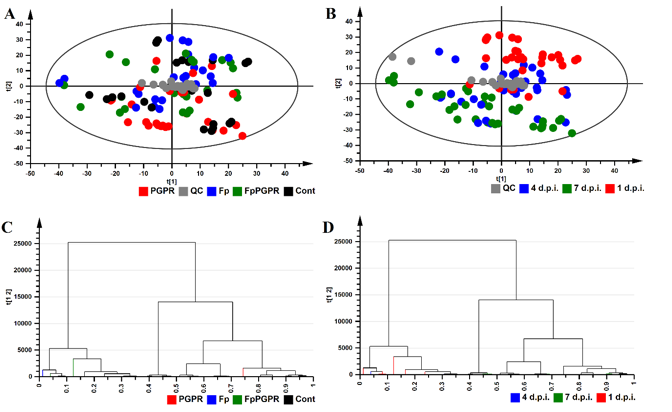

Supplement: Supplementary file 1 [file metabolites-09-00150-s001.zip › Supplementary material/Figure S8.tiff]

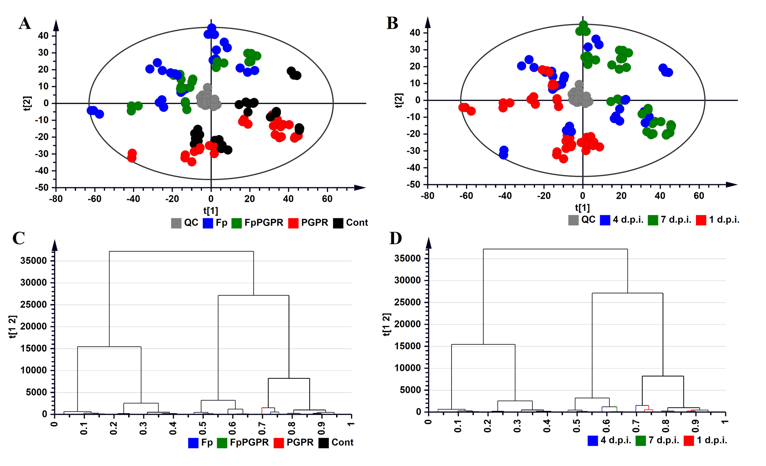

Supplement: Supplementary file 1 [file metabolites-09-00150-s001.zip › Supplementary material/Figure S4.tiff]

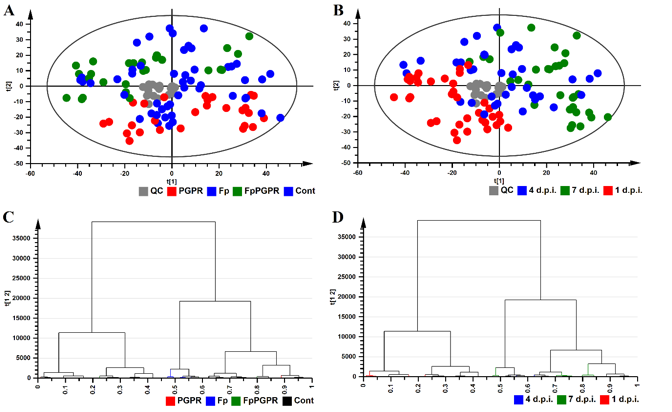

Supplement: Supplementary file 1 [file metabolites-09-00150-s001.zip › Supplementary material/Figure S7.tiff]

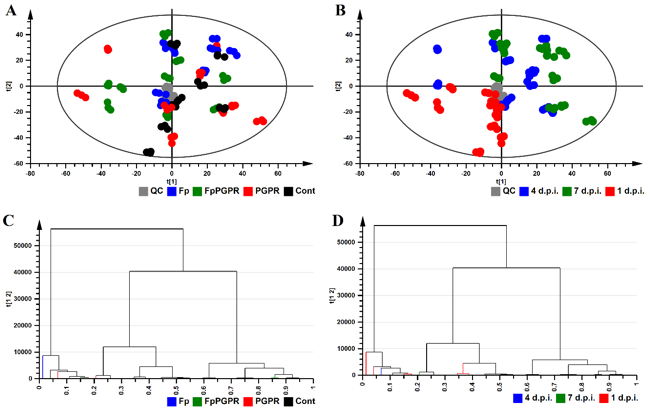

Supplement: Supplementary file 1 [file metabolites-09-00150-s001.zip › Supplementary material/Figure S6.tiff]

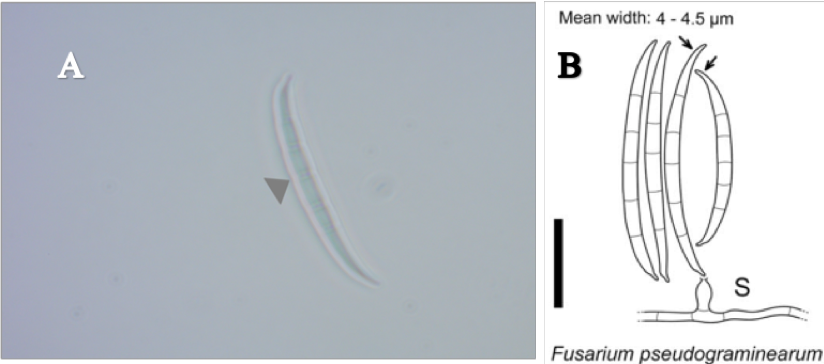

Supplement: Supplementary file 1 [file metabolites-09-00150-s001.zip › Supplementary material/Figure S1.tiff]

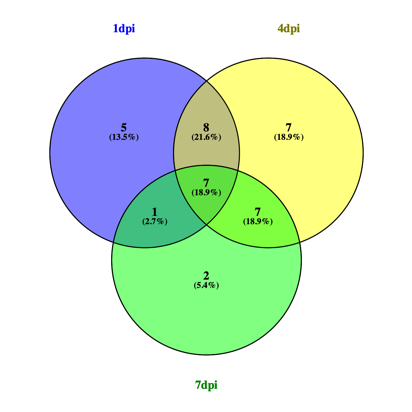

Supplement: Supplementary file 1 [file metabolites-09-00150-s001.zip › Supplementary material/Figure S12.tiff]
